# Systemic *Erysipelothrix rhusiopathiae* Infection with Rhomboid Skin Lesions in a Free-Ranging Short-Beaked Common Dolphin (*Delphinus delphis*) Stranded in Jeju, Republic of Korea: Post-Mortem Computed Tomography, Pathology and Multi-Tissue Molecular Detection

**DOI:** 10.3390/vetsci13101125

**Published:** 2026-10-21

**Authors:** Ji-Hyung Park, Yu Ri Heo, Young Jin Ryu, Ji Yeong Choi, Mohan Huang, Seyoung Lee, Jong-pil Seo, Adams Hei Long Yuen, Byung Yeop Kim, Seung Hyeok Seok, Sang Wha Kim

**Affiliations:** 1 College of Veterinary Medicine and Institute of Veterinary Science, Kangwon National University, Chuncheon 24341, Republic of Korea; mung90@kangwon.ac.kr (J.-H.P.);; 2 Department of Microbiology and Immunology, and Institute of Endemic Disease, College of Medicine, Seoul National University, Seoul 03080, Republic of Korea; 3 School of Medical and Health Sciences, Tung Wah College, Homantin, Kowloon, Hong Kong SAR, China; 4 College of Veterinary Medicine and Veterinary Medical Research Institute, Jeju National University, Jeju 63243, Republic of Korea; 5 Department of Marine Industrial and Maritime Police, College of Ocean Science, Jeju National University, Jeju 63243, Republic of Korea; 6 Department of Biomedical Sciences, College of Medicine, Seoul National University, Seoul 03080, Republic of Korea; 7 Cancer Research Institute, Seoul National University, Seoul 03080, Republic of Korea; 8 Institute of Endemic Diseases, Seoul National University Medical Research Center, Seoul 03080, Republic of Korea

**Keywords:** *Erysipelothrix rhusiopathiae*, erysipelas, *Delphinus delphis*, cetacean stranding, virtopsy, post-mortem computed tomography, 16S rRNA, septicaemia, Jeju, Republic of Korea

## Abstract

*Erysipelothrix rhusiopathiae* causes sporadic, often fatal septicaemia in cetaceans; published cases rest almost entirely on cultured isolates, and none has included post-mortem imaging. We investigated a juvenile male short-beaked common dolphin (*Delphinus delphis*) stranded on Jeju Island, Republic of Korea, using post-mortem computed tomography (PMCT) of the frozen carcass, necropsy, histopathology and tiered molecular testing. Rhomboid skin lesions extended through the skin into the blubber. PMCT documented gravity-dependent pulmonary opacification, fluid in the trachea and bronchi, peritoneal effusion, no decomposition gas and a gastric fishing hook, confirmed at necropsy. Culture was not attempted because the carcass had been stored frozen. *Erysipelothrix* spp. DNA was detected by broad-range 16S rRNA sequencing in 11 of 12 tissues, and *E. rhusiopathiae* was identified by species-specific PCR and amplicon sequencing in all 12 tissues, yielding one identical 356-bp sequence. Histopathology showed epidermal coagulative necrosis with septal panniculitis and abundant Gram-positive bacteria throughout the lesion, pulmonary haemorrhage and oedema, interstitial nephritis and reactive lymphadenitis, consistent with fatal septicaemia. The case provides imaging correlates of erysipelas, a culture-independent diagnostic framework for frozen carcasses and a tissue-by-tissue molecular dataset; the relationship of the strain to other Korean cetacean isolates remains to be established genomically.

## Simple Summary

*Erysipelothrix rhusiopathiae* is a bacterium best known for causing erysipelas in pigs, and it can also infect people who handle animals or fish. It kills dolphins and whales too, but such cases are rarely confirmed in the wild because stranded carcasses are often decomposed or frozen and the bacterium cannot be grown. We describe a young common dolphin found dead on Jeju Island, Republic of Korea, with the diamond-shaped skin sores typical of this disease. Because the body had been frozen we did not attempt culture; instead, we combined whole-body computed tomography scanning before dissection, a full post-mortem examination, microscopy of the organs and genetic testing of twelve tissues. The scan showed fluid-filled lungs and a swallowed fishing hook, and genetic testing found the bacterium in all twelve tissues, showing the infection had spread through the whole body and caused death. This is the first confirmed case in a common dolphin in the north-west Pacific and the first in a dolphin or whale examined by scanning before dissection. It shows how a reliable diagnosis can be reached in a frozen carcass without culture, and reminds people handling stranded marine mammals that this bacterium is a health risk to people.

## 1. Introduction

*Erysipelothrix rhusiopathiae* is a facultatively anaerobic, non-spore-forming, Gram-positive bacillus of the family Erysipelotrichaceae with a broad host range that includes swine, poultry, marine fish and mammals, and humans, in whom it causes erysipeloid [1,2]. Its pathogenesis has been characterised mainly in swine and in murine models [1,3]. After mucosal or cutaneous invasion, the bacterium disseminates haematogenously. Virulence depends on a hyaluronic-acid-containing capsule, which confers resistance to phagocytosis and allows survival within macrophages; on the surface protective antigen SpaA and the adhesins RspA and RspB, which mediate attachment to host cells and to extracellular-matrix components; and on secreted neuraminidase (sialidase), hyaluronidase and phospholipases, which cleave sialic acid and hyaluronan on endothelial and connective-tissue surfaces and facilitate vascular injury and tissue spread [1,2,3]. Neuraminidase-mediated damage to the vascular endothelium, with fibrin deposition, platelet aggregation and microthrombosis, underlies the characteristic lesions of acute erysipelas: widespread congestion, haemorrhage and oedema, serosanguineous body-cavity effusions and lymphadenopathy and, in the skin, ischaemic infarction of the territories supplied by thrombosed dermal arterioles, which produces the geometric “diamond-skin” plaques [1,2]. Death in the acute form results from septic shock and disseminated intravascular coagulation, whereas animals that survive the acute phase may develop chronic valvular endocarditis or polyarthritis [1,2]. In cetaceans, infection classically presents in two forms: an acute septicaemic form that is frequently fatal within hours to a few days, often with few or no premonitory signs, and a subacute dermatological form characterised by pathognomonic rhomboid skin plaques, which may resolve or progress to septicaemia [4,5]. Erysipelas was recognised in dolphins under human care in the 1960s and was for decades regarded as one of the most important causes of acute death in captive bottlenose dolphins, prompting the use of bacterins in some facilities; in free-ranging odontocetes the septicaemic form predominates and the disease is usually diagnosed only at necropsy [4,5]. The organism persists in the mucous slime of marine fish and in marine sediments and can be recovered from a substantial proportion of seafood samples [1,6], and in cetaceans infection is thought to be acquired through the ingestion of contaminated fish, with the oral and gastric mucosa as the presumed portal of entry, or through cutaneous inoculation of skin wounds [4,5].

Erysipelas has been documented in captive and free-ranging odontocetes worldwide, including bottlenose dolphins (*Tursiops truncatus*) [5,7,8], Atlantic spotted dolphins (*Stenella frontalis*) [8], a Pacific white-sided dolphin (*Lagenorhynchus obliquidens*) [9], harbour porpoises (*Phocoena phocoena*) and a short-beaked common dolphin (*Delphinus delphis*) in UK waters [10], a bottlenose dolphin in Brazil [11], a cluster of three bottlenose dolphins in San Diego in 2022 [12], a mass mortality of harbour porpoises in the Netherlands in 2021 that was attributed to two clonal lineages of *E. rhusiopathiae* by whole-genome sequencing of isolates [13], a bottlenose dolphin with predominant brain involvement in the western Mediterranean [14] and, in Korea, a rough-toothed dolphin (*Steno bredanensis*) stranded in Yeosu in 2018 [15]. In several of these cases, including the only previous common dolphin case and the Korean case, rhomboid skin lesions were absent or were not recorded, illustrating that gross lesions can be subtle or absent and that a laboratory diagnosis is required [8,10,12,13,15]. Isolation by culture remains the reference method, and in all but one of the free-ranging cases listed in Table 1 the diagnosis and any strain characterisation rested on a cultured isolate; in stranded animals, however, culture is frequently compromised by autolysis, freezing or post-mortem overgrowth, and molecular methods targeting the 16S rRNA gene or species-specific loci are increasingly used to establish the diagnosis and to map tissue distribution [8,11,15,16]. Stranding networks are, moreover, often obliged to store carcasses frozen before a full examination can be organised, and freezing and autolysis degrade tissue architecture, reduce the recovery of fastidious bacteria and make post-mortem overgrowth difficult to separate from true infection, so that diagnostic workflows that remain informative in frozen or partly autolysed carcasses are a practical need for stranding programmes [17]. To our knowledge, none of these reports included post-mortem imaging, and, apart from the Dutch porpoise mortality event [13], no sequence-level comparison between *E. rhusiopathiae* from different cetacean cases in the same region has been reported.

**Table 2:** Tissue-by-tissue molecular results for *Erysipelothrix rhusiopathiae* in the short-beaked common dolphin SL26-0209A.

Tissue	Broad-Range 16S rRNA PCR and Sequencing (91E/13B) ^a^	Genus-Directed PCR (MO101/MO102, 407 bp)	MO Amplicon Sequence (369 bp) ^b^	Species-Directed PCR (ER1F/ER1R, 400 bp)	ER1 Amplicon Sequence (356 bp) ^c^	GenBank Accession
Skin lesion 1 (lumbar flank)	+; mixed chromatogram; best match *Erysipelothrix*	+	Identical (335 bp recovered)	+	Identical	none
Skin lesion 2 (tail peduncle)	+; mixed chromatogram; best match *Erysipelothrix*	+	Identical	+	Identical	PZ956008 (MO); PZ983794 (ER1)
Cardiac blood	+; 434 bp; 434/434 (100%) ATCC 19414	+	Identical	+	Identical	PZ953691 (16S)
Lung	+; 434 bp; 434/434 (100%)	+	Identical	+	Identical	PZ953692 (16S)
Liver	+; 434 bp; 434/434 (100%)	+	Identical	+	Identical	none
Spleen	+; 387 bp; 387/387 (100%)	+	Identical	+	Identical	none
Kidney	+; 434 bp; 434/434 (100%)	+	Identical	+	Identical	none
Pancreas	+; 434 bp; 434/434 (100%)	+	Identical	+	Identical	none
Thyroid	+; 434 bp; 434/434 (100%)	+	Identical	+	Identical	none
Superficial cervical lymph node	+; 397 bp; 397/397 (100%)	+	Identical	+	Identical	none
Marginal lymph node	+; 415 bp; 415/415 (100%)	+	Identical	+	Identical	none
Skeletal muscle	− (mixed chromatogram; no *Erysipelothrix* sequence resolved)	+	Identical	+	Identical	none
No-template control	−	−	n.a.	−	n.a.	none
Extraction blank ^d^	−	−	n.a.	−	n.a.	none
Uninfected dolphin tissue ^d^	n.t.	−	n.a.	−	n.a.	none
*Escherichia coli* DH5α DNA ^d^	n.t.	−	n.a.	−	n.a.	none

+, amplicon of the expected size; −, no amplicon; n.a., not applicable; n.t., not tested. ^a^ Contig length after removal of the primer sequences (query coverage 100% for all contigs), and identity to the 16S rRNA gene of the *E. rhusiopathiae* type strain ATCC 19414 (NR_040837.1); this region does not discriminate *E. rhusiopathiae* from *E. tonsillarum* and other *Erysipelothrix* species, and the results are therefore reported as detection of *Erysipelothrix* spp. ^b^ Identical to the 16S rRNA gene of ATCC 19414 and to the corresponding region of other *Erysipelothrix* species (genus-level target). ^c^ Identical to the sequence deposited as PZ983794 (353/356 to ATCC 19414; 354/356 to KC-Sb-R1). ^d^ Controls included in the confirmatory run performed during revision, in which DNA was re-extracted from all 12 tissues (Supplementary Figure S3); all 12 re-extracted tissues were again positive with both assays.

The short-beaked common dolphin is widely distributed in temperate and tropical waters; in Korean waters it occurs predominantly in the East Sea, where it is the most frequently bycaught delphinid, and strandings on the southern coast and Jeju Island are comparatively uncommon [18,19]. Of 455 cetacean carcasses recorded as stranded or bycaught in the waters around Jeju Island between 2016 and 2023, only seven (1.5%) were common dolphins (one each in 2017, 2020 and 2023, and two each in 2019 and 2021), compared with the narrow-ridged finless porpoise, which accounts for the great majority of records. Common dolphin carcasses have, however, become more frequent recently: of 102 cetacean carcasses recorded between January 2024 and July 2026, 11 (10.8%) were common dolphins, all recovered between October and February (three in December 2024, four between October and December 2025, and four between January and February 2026, including the animal described here), and pods of 50–100 common dolphins were sighted off the south-western coast of Jeju in November 2024 and January 2025 (Jeju stranding records database 2014–2026, Whale–Marine Life Conservation Research Center, Gidang Institute of Marine Science, Jeju National University; unpublished data; Supplementary Table S3). Post-mortem investigation of stranded marine animals in Korea has expanded in recent years, and our group has adopted a “virtopsy” approach in which whole-body computed tomography (CT) is performed before conventional necropsy to document lesions non-invasively, to detect foreign bodies, skeletal abnormalities and gas, and to guide sampling; this protocol has been applied to a long-term monitored Indo-Pacific bottlenose dolphin (*Tursiops aduncus*) [20], to vessel-strike trauma in spotted seals (*Phoca largha*) [21], to CT-based craniofacial morphometry in sharks [22], and to vertebral deformities in narrow-ridged finless porpoises (*Neophocaena asiaeorientalis*) [23].

Here we describe a case of disseminated *E. rhusiopathiae* infection in a free-ranging common dolphin stranded on Jeju Island, investigating a frozen carcass with a protocol that integrated PMCT, gross and histopathological examination and multi-tissue molecular detection. Beyond extending the known host and geographic range of the disease to *D. delphis* in the Northwest Pacific, the case contributes three elements that are absent from previous reports (Table 1): (i) a description of PMCT findings in cetacean erysipelas, which to our knowledge has not been reported, and their correlation with necropsy and histopathology; (ii) a culture-independent, tiered molecular workflow in which broad-range 16S rRNA sequencing, genus- and species-directed PCR and amplicon sequencing were applied to 12 tissues of a frozen carcass, with the species assignment resting on the sequence of a species-discriminating locus rather than on 16S rRNA data alone; and (iii) a tissue-by-tissue molecular dataset in which the sequence obtained directly from tissue is compared with the genome of the only other *E. rhusiopathiae* isolate from a Korean cetacean and with all publicly available *E. rhusiopathiae* genome assemblies. We also place the case in the context of a recent increase in the recorded proportion of common dolphins among cetacean carcasses around Jeju documented by the regional stranding network.

## 2. Case Description

### 2.1. Stranding History and Carcass Handling

On 11 January 2026, a dead common dolphin was found stranded on the shore at Changcheon-ri, Andeok-myeon, Seogwipo-si, Jeju Island, Republic of Korea (adjacent to Daepyeong Port; approx. 33.24° N, 126.36° E). The carcass was discovered by a member of the public, who reported it to the Korea Coast Guard; the report was relayed to Jeju National University at 16:24 on the same day, and the animal was identified in the field and taken into custody by the university stranding team. The carcass was stored frozen (−20 °C). The frozen animal was transported to the Jeju National University Equine Hospital for post-mortem CT (PMCT) scanning on 9 February 2026 and, after thawing, was necropsied at Korea Fisheries Resources Agency (FIRA) Jeju branch on 13 February 2026 (case ID SL26-0209A). Because the carcass had been frozen, bacterial culture was not attempted and the aetiological investigation relied on molecular methods. All specimens were collected from one naturally deceased animal, and sample collection was conducted under a marine protected species collection permit approved by the Governor of Jeju Special Self-Governing Province. This study followed all applicable ethical guidelines for wildlife research in the Republic of Korea.

### 2.2. Animal Identification and Morphometrics

**Figure 1:**
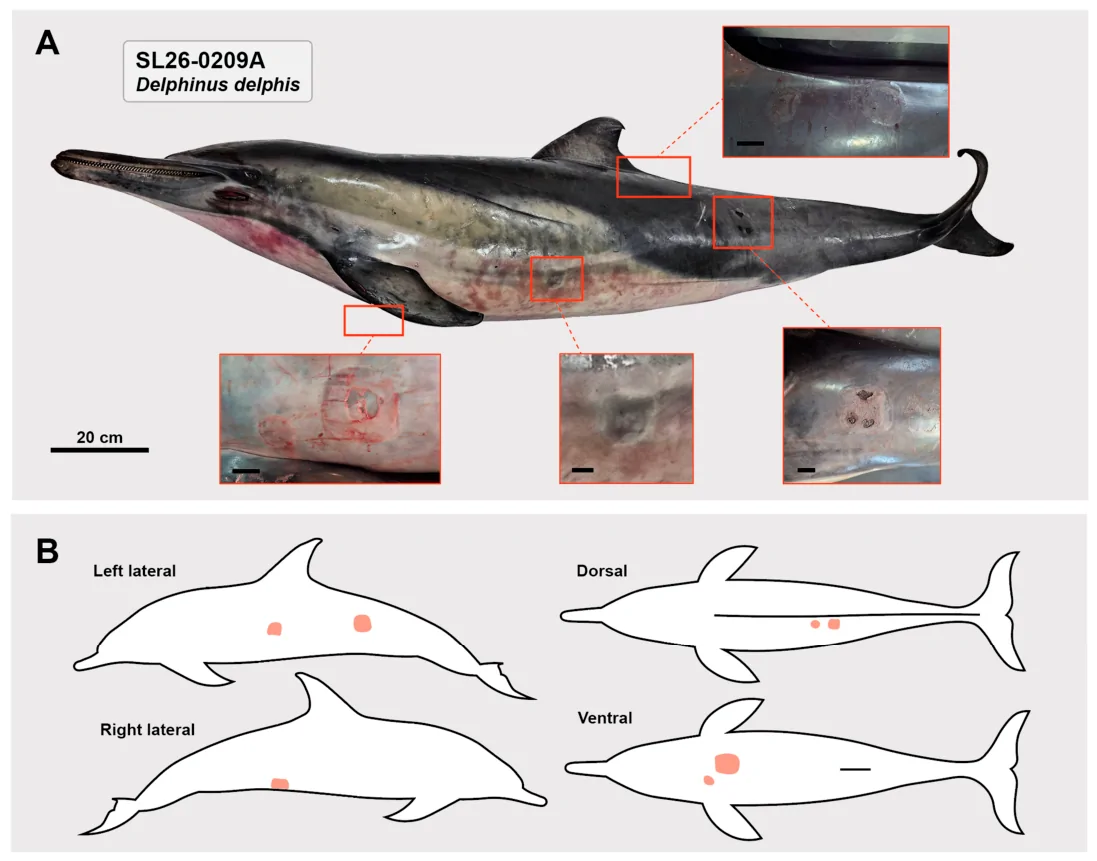
Gross appearance and distribution of skin lesions in the short-beaked common dolphin (*Delphinus delphis*, SL26-0209A) with systemic *Erysipelothrix rhusiopathiae* infection. (**A**) Left lateral view of the carcass (scale bar, 20 cm) with insets showing quadrangular-to-rhomboid skin lesions with ulcerated centres on the flank, peduncle, dorsal ridge and ventral thorax; each inset carries its own scale bars (2 cm). (**B**) Schematic distribution of the lesions (shaded) on the left lateral, right lateral, dorsal and ventral body surfaces.

The animal was identified in the field as a delphinid with a long rostrum, a dark dorsal cape and a pale tan thoracic patch forming the criss-cross (hourglass) flank pattern typical of the genus (Figure 1A). Species identity was confirmed by mitochondrial DNA sequencing. The near-complete cytochrome b gene was amplified with the primers CB-out1 (5′-AATGAYATGAAAARYCATCGTTG-3′) and CB-out2 (5′-TCTTCCTTGAGTCTTAGGGAG-3′) and sequenced with the amplification primers and the internal primers CB-in1 (5′-TTRTTRGATCCTGTTTCRTG-3′) and CB-in2 (5′-TGAGGACAAATATCATTYTGAG-3′) [24] (94 °C for 5 min; 32 cycles of 94 °C for 1 min, 55 °C for 1 min and 72 °C for 1 min; 72 °C for 5 min), and a fragment of the 5′ end of the control region (D-loop) was amplified and sequenced with the primers Dlp1.5 (5′-TCACCCAAAGCTGRARTTCTA-3′) and Dlp5 (5′-CCATCGWGATGTCTTATTTAAGRGGAA-3′) [25,26,27], using a touch-down profile [28] (93 °C for 3 min; 9 cycles of 93 °C for 45 s, annealing for 45 s at 63 °C decreasing by 1 °C per cycle to 55 °C, and 72 °C for 1 min; 27 cycles of 93 °C for 45 s, 53 °C for 45 s and 72 °C for 1 min; and a final extension at 72 °C for 10 min). Sanger sequencing was performed by Macrogen (Seoul, Republic of Korea). A 1143-bp cytochrome b contig (four reads; codons 3–380 of the 1140-bp gene plus the 5′ end of tRNA-Thr) and a 480-bp D-loop contig (two reads; primer sequences and a low-quality 3′ terminal segment removed) were assembled and compared with GenBank by BLASTn (megablast; BLAST+ 2.17.0, NCBI web server)against the NCBI core nucleotide database. The cytochrome b contig was identical (1143/1143 bp) to the complete mitogenome of a *Delphinus delphis* specimen from Korean waters (voucher CRI007520, GenBank MH000365.1), followed by other *D. delphis* mitogenomes (99.5–99.7%), and was separated from the eastern Pacific long-beaked common dolphin *D. bairdii* (≤98.9%) and from complete mitogenomes of *Stenella* spp. (≤97.9%). The D-loop contig showed 99.8% identity (479/480 bp) to the CRI007520 mitogenome and was identical over the full length of the record (465/465 bp) to the control-region haplotype KH14 of *D. delphis* from the East Sea, Republic of Korea (KY212898.1); 96 of the 100 best hits were Delphinus records. The best full-coverage *Stenella* hit was 98.8% identical (AY168600.1), but two control-region records deposited as *Stenella coeruleoalba* in 2001 (AY046546.1 and AY046548.1) were also identical to the contig and to haplotype KH14, so the control region alone would not have excluded *S. coeruleoalba* and the species assignment rests on cytochrome b (Supplementary Table S2). The cytochrome b and control-region sequences were deposited in GenBank under accession numbers PZ946319 and PZ946320, respectively (the PZ946320 record contains the complete 499-bp control-region assembly, of which the 480-bp trimmed segment described above was used for the BLAST+ 2.17.0 comparison). The animal was male. Total body length, measured in a straight line from the tip of the rostrum to the fluke notch after thawing, was 214.3 cm (a provisional field measurement of 220 cm, taken along the body contour at recovery and recorded in the stranding database, is listed in Supplementary Table S3), and body weight was 92 kg. Tooth counts were 44/45 (upper left/right) and 47/45 (lower left/right). Body condition was assessed as good, with dorsal, lateral and ventral blubber thickness at the level of the dorsal fin of 9, 10 and 7 mm, respectively. The presence of marginal lingual papillae and the body length indicated a juvenile animal. The carcass was in fresh to slightly decomposed condition (Smithsonian code 2).

### 2.3. Post-Mortem Computed Tomography (Virtopsy)

**Figure 2:**
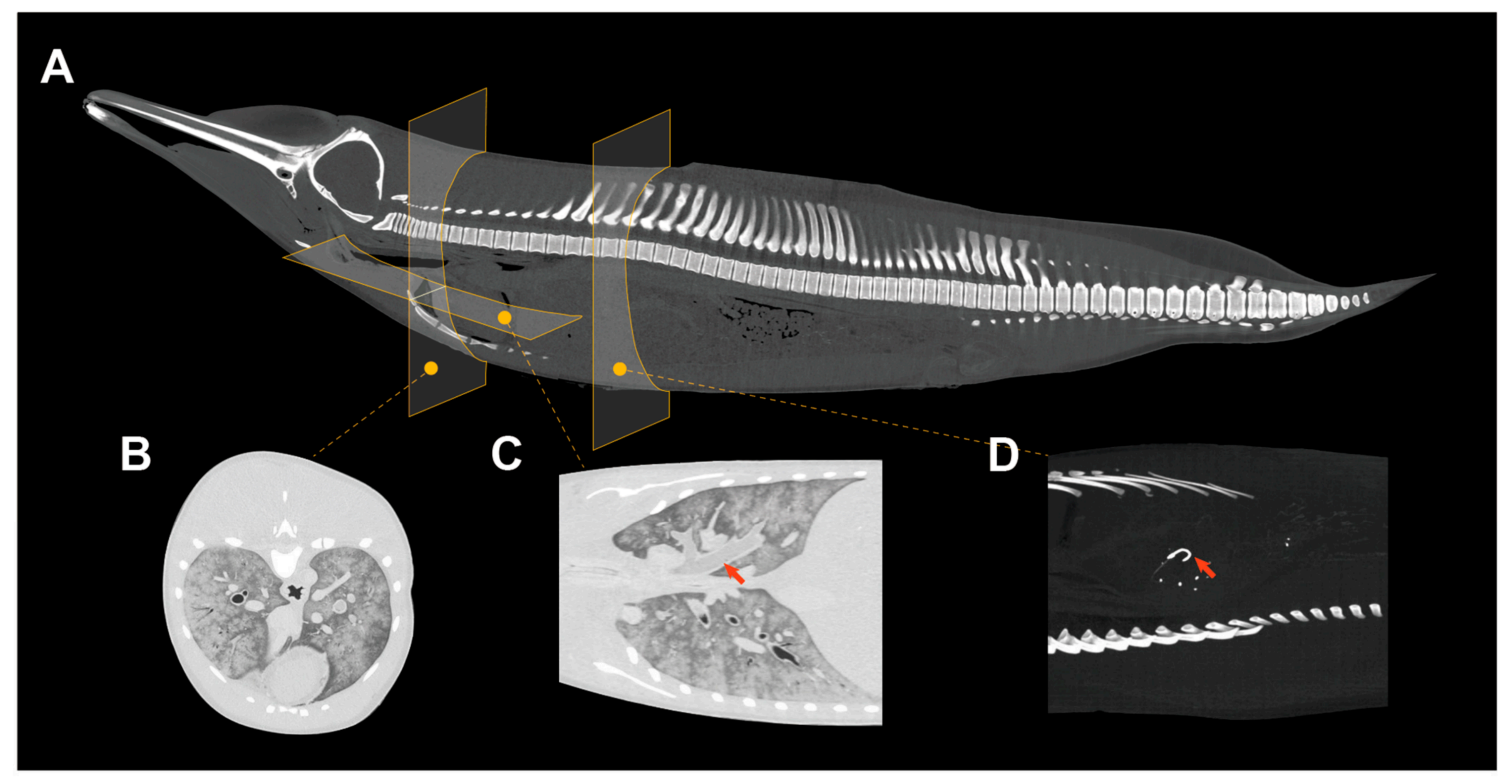
Post-mortem computed tomography of the short-beaked common dolphin (*Delphinus delphis*, SL26-0209A) with systemic *Erysipelothrix rhusiopathiae* infection. (**A**) Whole-body sagittal reconstruction stitched from three overlapping acquisitions, with the positions of the lower panels indicated. (**B**) Transverse image of the thorax (lung window) showing bilateral heterogeneous ground-glass to consolidative opacity with a gravity-dependent gradient. (**C**) Coronal reconstruction of the lungs showing the same pattern with fluid in the principal bronchi (arrow). (**D**) Lateral reconstruction of the abdomen (bone window) showing a curvilinear metallic foreign body (fishing hook; arrow).

Whole-body PMCT was performed on 9 February 2026 at the Jeju National University Equine Hospital, with the carcass still frozen, using a 16-row, 32-slice helical CT scanner (Aquilion Lightning, Canon Medical Systems, Otawara, Japan). Because the total body length exceeded the scannable range and the highly variable body circumference could affect image quality, the carcass was imaged in three overlapping acquisitions: rostrum to abdomen (916 mm), abdomen to caudal peduncle (973 mm), and peduncle to flukes (862 mm). Acquisition parameters included 100 kVp, 250 mA, and a 0.75 s rotation time. The scanned field of view was set to 390 mm for the cranial–abdominal and abdominal–caudal peduncle scans, and 306 mm for the peduncle–fluke scan to closely fit the animal’s girth, yielding a 512 × 512 matrix (in-plane pixel size 0.60–0.76 mm). Images were reconstructed at 1.0-mm slice thickness on the native scanner console (Canon Medical Systems) using the FC13 (soft-tissue) and FC30 (bone) kernels, and were reviewed on a Horos workstation (version 4.0.0, Horos Project) in lung (W 1500/L −600), soft-tissue (W 400/L 40) and bone (W 3000/L 500) windows with multiplanar reconstruction and maximum-intensity projection (Figure 2). The images were read independently by two readers experienced in cetacean PMCT (A.H.L.Y. and S.W.K., with >10 years of experience in marine-mammal PMCT), and the findings were reconciled by consensus before necropsy.

In the thorax, both lungs showed extensive, heterogeneous increase in attenuation with a gravity-dependent gradient, the dependent lung was consolidated, whereas the non-dependent lung showed patchy ground-glass opacity and only small residual areas of normal parenchyma. Fluid attenuation was present within the lumen of the trachea and both principal bronchi. A pleural effusion could not be delineated with confidence from the dependent, consolidated lung and chest wall on the non-contrast images of the frozen carcass.

The stomach compartments were distended by fluid and a moderate amount of gas, and a small curvilinear, markedly hyperattenuating (>3000 HU) metallic object approximately 30 mm in length with streak artefact was identified within the lumen of the fundic stomach compartment, suggestive of fishing hook (Figure 2D). A thin rim of fluid attenuation was present along the dependent peritoneal surface. Gas was confined to the gastrointestinal lumen, with no intravascular, subcutaneous or intra-organ gas.

### 2.4. Gross Findings

Externally, multiple sharply demarcated, quadrangular to rhomboid skin lesions with raised margins and depressed centres, measuring 3–8 cm in greatest dimension, were observed at several anatomical locations: one on the left flank, one on the left lateral aspect of the peduncle, one on the right lateral aspect of the peduncle, two along the dorsal ridge, and two in the ventral thoracic region (Figure 1). Lesion centres were irregularly ulcerated and dark red to black, with a pale, slightly raised rim, and on the blubber surface the lesions were mirrored by well-circumscribed, square-to-rectangular, grey-to-tan areas of discolouration extending through the full thickness of the epidermis and dermis into the blubber (Figure 1A, insets). Several smaller, healing lesions were also present. Marginal lingual papillae were present. Cervical lymph nodes were found enlarged. The thoracic cavity contained sanguineous pleural fluid; the lungs were diffusely wet, orange-tan and spongy, and exuded fluid on section. Sanguineous fluid was also present in the peritoneal cavity. The spleen was accompanied by two accessory spleens. The forestomach contained brownish fluid and numerous nematodes embedded in the wall (several of which were collected for molecular identification, Section 2.6); the fundic stomach contained a corroded fishing hook, corresponding to the metallic foreign body identified on CT, lodged in the mucosa; the surrounding gastric wall was markedly thickened and coated with tenacious mucus, gross changes suggestive of a chronic foreign-body gastritis and indicating that the hook had been retained for a prolonged period before death; the pyloric stomach contained malodorous yellowish fluid. The heart, liver, kidneys, adrenal glands and reproductive tract showed no gross lesions. The left rib cage comprised 14 ribs plus one floating rib.

### 2.5. Histopathology

**Figure 3:**
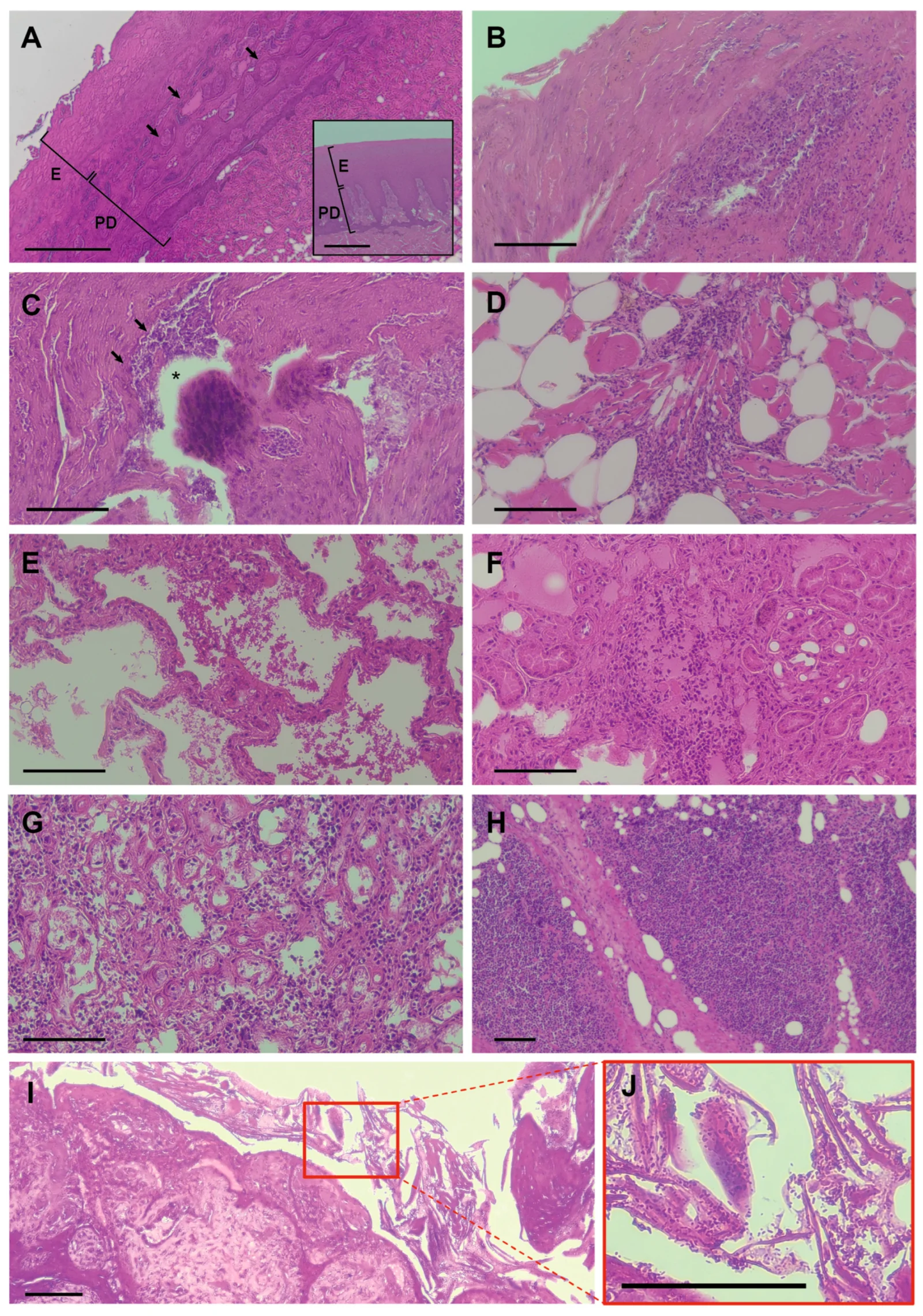
Histopathology of the short-beaked common dolphin (*Delphinus delphis*, SL26-0209A) with systemic *Erysipelothrix rhusiopathiae* infection. (**A**) Rhomboid skin lesion, low magnification. The epidermis is diffusely hypereosinophilic with loss of nuclear detail, and the dermal papillae (arrows) are likewise necrotic; coagulative necrosis has effaced the normal architecture of the epidermis (E) and papillary dermis (PD) (brackets). Inset: non-lesional trunk skin from the same animal at the same magnification, showing an intact epidermis (E) and regularly spaced dermal papillae (PD). (**B**) Deeper dermis of the lesion: hypereosinophilic, fragmented collagen infiltrated by dense sheets of degenerate neutrophils with abundant karyorrhectic debris. (**C**) Margin of an ulcerated area: the residual necrotic epidermis is separated from the dermis by a subepidermal cleft (asterisk) that contains a large basophilic, granular aggregate morphologically consistent with a bacterial colony; the adjacent dermis is bordered by a band of degenerate neutrophils (arrows). (**D**) Superficial blubber immediately beneath the dermis of the lesion. The fibrous septa and adjacent adipose tissue are necrotic, with hypereosinophilic, fragmented collagen bundles infiltrated by neutrophils and macrophages (septal panniculitis). (**E**) Lung: congested alveolar septa with alveolar haemorrhage and proteinaceous oedema fluid. (**F**) Kidney: interstitial infiltrate of lymphocytes, plasma cells and neutrophils with interstitial oedema and tubular epithelial degeneration. (**G**) Marginal lymph node: dilated sinuses containing proteinaceous fluid, histiocytes and neutrophils. (**H**) Spleen: a prominent lymphoid follicle (right) adjacent to a fibrous trabecula, with congested, hypercellular red pulp (left). (**I**) Rhomboid skin lesion, Gram stain, low magnification: numerous Gram-positive bacteria (purple) throughout the necrotic epidermis and the underlying dermis, extending well beyond the ulcerated surface; the boxed area is enlarged in (**J**). (**J**) Dense clusters of Gram-positive bacilli within the necrotic epidermis and in a subepidermal cleft, with scattered organisms along the fragmented epidermal lamellae. (**A**–**H**), haematoxylin and eosin; (**I**,**J**), Gram stain (crystal violet with safranin O counterstain). Original magnification: (**A**) and inset, ×40; (**B**–**G**), ×200; (**H**), ×100; (**I**), ×100; (**J**), ×200. E, epidermis; PD, papillary dermis. Scale bars: (**A**) and inset, 500 µm; (**B**–**G**), 100 µm; (**H**), 200 µm; (**I**), 100 µm; (**J**), 200 µm.

Tissue samples of the skin lesions (lesion centre, lesion margin and adjacent non-lesional skin), lung, heart, liver, spleen, kidney, adrenal gland, pancreas, thyroid, superficial cervical and marginal lymph nodes, fundic stomach, intestine, brain and gonad were collected at necropsy with a fresh blade for each organ, fixed in 10% neutral buffered formalin for at least 48 h at room temperature, trimmed to a thickness of ≤5 mm and processed routinely (graded ethanol, xylene, paraffin infiltration) in an automated tissue processor; no decalcification was required. Eleven paraffin blocks were prepared, each block was sectioned at 4 µm, and one section per block (11 sections in total) was stained with haematoxylin and eosin by a standard regressive protocol (deparaffinisation in xylene and rehydration through graded ethanol; Harris haematoxylin, 5 min; differentiation in 1% acid alcohol; bluing in Scott’s tap water substitute; 1% alcoholic eosin Y, 1 min; dehydration through graded ethanol, clearing in xylene and mounting in synthetic resin). All 11 sections were examined in their entirety by light microscopy and were read independently by two veterinarians (S.W.K. and J.-H.P.); discrepancies were resolved by joint review at a multi-head microscope, and the fields shown in Figure 3 were selected as representative of the lesions described below. Serial sections of the skin lesion were additionally stained with a commercial Gram staining set (Gram Staining Set, cat. no. 156200; Biozoa, Seoul, Republic of Korea) adapted to paraffin sections (crystal violet, 1 min; Gram’s iodine, 1 min; the set’s decolourising solution for 5, 10 or 15 s on parallel sections; safranin O counterstain, 60 s), the 15 s decolourisation giving the clearest discrimination; the bacterial aggregates on the ulcer surface served as an internal control for staining. Histological examination was hampered to a moderate degree by freeze–thaw artefact (ice-crystal clefts and loss of nuclear detail), but the lesions were interpretable. In the rhomboid skin lesions, the epidermis was diffusely hypereosinophilic with loss of nuclear detail (coagulative necrosis) and focally ulcerated, and the dermal papillae and superficial dermis were likewise necrotic and infiltrated by degenerate neutrophils and mononuclear cells with abundant karyorrhectic debris (Figure 3A), in contrast to the intact epidermis and regularly spaced dermal papillae of non-lesional trunk skin (Figure 3A, inset). In the deeper dermis, the collagenous stroma was hypereosinophilic and fragmented and was infiltrated by dense sheets of degenerate neutrophils with abundant karyorrhexis (Figure 3B). At the margin of the ulcerated areas, the residual necrotic epidermis was separated from the dermis by a subepidermal cleft containing degenerate neutrophils and cellular debris, and large basophilic, granular aggregates morphologically consistent with bacterial colonies lay within these clefts and at the ulcer base, surrounded by a band of degenerate neutrophils (Figure 3C); because these aggregates were confined to the ulcerated surface, secondary colonisation of the ulcer by environmental bacteria could not be excluded on morphological grounds. The inflammation extended into the superficial blubber immediately beneath the dermis, where the fibrous septa and adjacent adipose tissue were necrotic, with hypereosinophilic, fragmented collagen bundles infiltrated by neutrophils and macrophages, consistent with a necrotising septal panniculitis (Figure 3D); involvement of the underlying skeletal muscle was not identified in the sections examined. In the lungs, alveolar septa were congested and alveoli contained abundant erythrocytes and proteinaceous oedema fluid with scattered fibrin strands, without a bronchopneumonic exudate (Figure 3E). The kidneys showed multifocal interstitial infiltrates of lymphocytes, plasma cells and neutrophils with interstitial oedema and tubular epithelial degeneration (Figure 3F). The marginal (mesenteric) and superficial cervical lymph nodes showed dilated subcapsular and medullary sinuses filled with proteinaceous fluid, histiocytes and neutrophils, with congestion and moderate cortical lymphoid depletion, consistent with acute reactive lymphadenitis (Figure 3G). The spleen was congested, with prominent lymphoid follicles and periarteriolar lymphoid sheaths and a red pulp rich in neutrophils (Figure 3H). The sections of the fundic stomach did not include the site of the fishing hook; the markedly thickened wall around the hook was therefore not examined histologically, and the gastritis is described on gross grounds only. No significant microscopic lesions, other than congestion and freeze–thaw artefact, were identified in the heart, liver, adrenal gland, pancreas, thyroid, stomach (away from the hook), intestine, brain or gonad. Bacterial emboli or intravascular bacteria, which have been described in multiple organs of septicaemic delphinids [8,10,12], were not identified in any organ in the haematoxylin and eosin sections; Gram staining, which readily demonstrated the organisms in the skin lesions (Figure 3I,J), was not performed on the other organs. Gram staining of the skin lesion showed large numbers of Gram-positive bacteria (slender bacilli, singly and in short chains) distributed throughout the necrotic epidermis and the papillary and deep dermis of the lesion, well beyond the ulcerated surface, with dense clusters within subepidermal clefts (Figure 3I,J).

### 2.6. Molecular Detection of Erysipelothrix rhusiopathiae

Genomic DNA was extracted from two skin lesions (lumbar flank and tail peduncle), cardiac blood, lung, liver, spleen, kidney, pancreas, thyroid, two lymph nodes (left superficial cervical and marginal) and skeletal muscle using the DNeasy Blood & Tissue Kit (Qiagen, Hilden, Germany) according to the manufacturer’s instructions. Each tissue was sampled at necropsy with a fresh sterile blade and forceps and placed in a separate tube; DNA extraction and PCR set-up were performed in a laboratory in which no *Erysipelothrix* culture, isolate or positive-control DNA had ever been handled, in a pre-PCR area physically separated from the post-PCR area, and with filter tips; an extraction blank was not included in the original extractions, and the confirmatory run described below was therefore performed with one. No reference strain of *E. rhusiopathiae*, including the Korean isolate KC-Sb-R1 [15], has been held in our laboratory; a positive control was therefore not included, and the identity of every amplicon was instead verified by bidirectional sequencing (below). A broad-range PCR targeting a ~500-bp region of the bacterial 16S rRNA gene was performed with primers 91E (5′-GGAATTCAAAKGAATTGACGGGGGC-3′) and 13B (5′-CGGGATCCCAGGCCCGGGAACGTATTCAC-3′) [29], and amplicons were sequenced bidirectionally by Sanger sequencing (Macrogen, Seoul, Republic of Korea). Sequences were compared with GenBank using BLASTn. *Erysipelothrix* 16S rRNA sequences were the top BLAST hits for the amplicons obtained from both skin lesions, cardiac blood, lung, liver, spleen, kidney, pancreas, thyroid and both lymph nodes (11 of 12 tissues), whereas the amplicon from skeletal muscle gave a mixed chromatogram in which no *Erysipelothrix* sequence could be resolved (Table 2). The contigs from cardiac blood, lung, kidney, liver, pancreas and thyroid (434 bp after removal of the primer sequences) and from the spleen and both lymph nodes (387–415 bp, shorter owing to read quality) were identical to each other and to the 16S rRNA gene of the *E. rhusiopathiae* type strain ATCC 19414 (GenBank NR_040837.1) over the sequenced region, and the amplicons from the two skin lesions gave mixed chromatograms consistent with a polymicrobial ulcer surface in which an *Erysipelothrix* sequence nevertheless remained the best match. Because this region of the 16S rRNA gene does not separate *E. rhusiopathiae* from *E. tonsillarum* and other closely related *Erysipelothrix* species (identity ≥99.5%), the broad-range results are reported as detection of *Erysipelothrix* spp. The sequences from cardiac blood and lung were deposited in GenBank under accession numbers PZ953691 and PZ953692. All extracts were then tested with an *Erysipelothrix* genus-directed PCR targeting the 16S rRNA gene (primers MO101, 5′-AGATGCCATAGAAACTGGTA-3′, and MO102, 5′-CTGTATCCGCCATAACTA-3′; 407 bp) [30] and with the *E. rhusiopathiae*-directed PCR of Takeshi et al. [31], which targets a non-coding region downstream of the 5S rRNA gene of the rRNA operon (positions 4119–4515 of GenBank AB019247; primers ER1F, 5′-GTTCATCTCTCTAATGCACTAC-3′, and ER1R, 5′-TGTTGGACTACTAATCGTTTCG-3′). The product is 399 bp in the strains examined in the original description and 400 bp in most publicly available *E. rhusiopathiae* genomes and in the present case, owing to a one-nucleotide difference in a homopolymer tract. Each 20 µL reaction (AccuPower PCR PreMix, Bioneer, Daejeon, Republic of Korea) contained 0.5 µM of each primer and 1 µL of template DNA. Both assays were run under the same conditions: 95 °C for 3 min; 40 cycles of 94 °C for 35 s, 55 °C for 40 s and 72 °C for 40 s; and 72 °C for 5 min, with a no-template control in each run. Amplicons of the expected size were obtained with both assays from all 12 samples, including skeletal muscle, in which the band was weaker than in the other tissues, whereas the no-template controls remained negative (Supplementary Figure S2). During revision, DNA was re-extracted from all 12 tissues and both assays were repeated with the following controls processed in parallel: an extraction blank carried through the entire extraction procedure, DNA from a tissue of an unrelated, uninfected dolphin extracted with the same kit, DNA of *Escherichia coli* DH5α, and a no-template control. All 12 tissue extracts were again positive with both assays, whereas the extraction blank, the uninfected-dolphin extract, the *E. coli* DNA and the no-template control remained negative; the uncropped gels with molecular-size ladders and the complete lane map are shown in Supplementary Figure S3 and the results are summarised in Table 2. Bidirectional sequencing of the ER1F/ER1R amplicons from all 12 tissues yielded an identical 356-bp sequence (excluding the primer-binding sites), and the MO101/MO102 amplicons from all 12 tissues yielded the same 16S rRNA sequence (369 bp in 11 tissues; 335 bp recovered from skin lesion 1, identical over its length), which was identical to that of ATCC 19414 but, as expected for this genus-level target, also to the corresponding region of *E. tonsillarum*, *E. piscisicarius*, *E. enhydrae* and *E. amsterdamensis*. In contrast, the ER1 sequence discriminated among the currently recognised species: it differed from the *E. rhusiopathiae* type strain ATCC 19414 and from 17 other complete *E. rhusiopathiae* genomes at three positions (353/356, 99.2% identity), was most similar (354/356, 99.4%) to the isolate KC-Sb-R1 from the rough-toothed dolphin stranded in Korea in 2018 [15], with which it shared one of the three substitutions, and showed 96.1–96.3%, 90.7% and 86.5% identity to the homologous regions of *E. amsterdamensis* and *E. enhydrae*, *E. piscisicarius* and *E. tonsillarum*, respectively; in the maximum-likelihood tree the sequence clustered with *E. rhusiopathiae* and was clearly separated from all other species (Supplementary Figure S1 and Table S1). An in silico re-assessment of the ER1F/ER1R primers against the species described since the assay was published showed no mismatches to 230 *E. rhusiopathiae* genome assemblies and 3–10 mismatches per primer to *E. tonsillarum*, *E. piscisicarius* and *Erysipelothrix* sp. HDW6A and D19-032, but only one or two mismatches per primer to *E. enhydrae* and *E. amsterdamensis* (Supplementary Table S1), so that a positive ER1 PCR alone would not exclude these two recently described species and the species assignment in this study rests on the amplicon sequence. To place the three substitutions in context, the ER1 region was extracted from all 237 *E. rhusiopathiae* assemblies available in the NCBI Assembly database (September 2026), 230 of which covered the three variable positions (Supplementary Table S4): the substitution shared with KC-Sb-R1 was also present in four assemblies from unrelated sources (pigs from Australia, Argentina and the USA, and a metagenome-assembled genome from the Democratic Republic of the Congo), whereas the other two substitutions were not found in any assembly, so that the dolphin sequence was distinct from every genome examined, including the 18 isolates from the 2021 Dutch harbour-porpoise mortality [13]. The public collection is, however, dominated by porcine isolates (170 of the 230 assemblies, 152 of them from a single Australian collection) and contains only three cetacean genomes (Supplementary Table S4). The MO101/MO102 and ER1F/ER1R sequences from the tail skin lesion were deposited in GenBank under accession numbers PZ956008 and PZ983794; the sequences from the other 11 tissues were identical to these and are provided, together with all chromatograms, in Supplementary Data S1. Chromatograms were quality-trimmed with the modified Mott algorithm (Phred cut-off 20) and the forward and reverse reads of each amplicon were merged by reference-guided pairwise alignment (match 2, mismatch −3, gap opening −6, gap extension −2; Biopython 1.88 PairwiseAligner) against the 16S rRNA gene of ATCC 19414 (NR_040837.1) or the ER1 region of KC-Sb-R1 (CP033601.1); conflicting base calls were resolved in favour of the higher Phred score and all homopolymer-length discrepancies were verified by visual inspection of the traces. BLASTn searches used megablast against the NCBI core nucleotide database (core_nt, September 2026). For the ER1 phylogeny, the amplicon and the homologous regions of the 33 reference sequences listed in Supplementary Table S1 were aligned with MAFFT v7.505 (L-INS-i), primer-binding sites and columns with >50% gaps were removed (356 aligned positions), and a maximum-likelihood tree was inferred with IQ-TREE 2.0.7 under the best-fit model selected by ModelFinder (HKY+F+G4 by Bayesian information criterion) with 1000 ultrafast bootstrap and 1000 SH-aLRT replicates, rooted on *E. larvae* as the most divergent member of the genus. Identical reference sequences were collapsed into single tips. The alignment, tree file and analysis scripts are provided in Supplementary Data S1.

To identify the gastric nematodes, genomic DNA was extracted from one of the worms collected from the forestomach wall with the same kit, and a fragment of the mitochondrial small-subunit rRNA gene (rrnS) was amplified with the primers MH3 (5′-TTGTTCCAGAATAATCGGCTAGACTT-3′) and MH4.5 (5′-TCTACTTTACTACAACTTACTCC-3′) [32] (94 °C for 5 min; 35 cycles of 94 °C for 30 s, 55 °C for 45 s and 72 °C for 90 s; 72 °C for 10 min) and sequenced bidirectionally. Each 20 µL reaction (AccuPower PCR PreMix, Bioneer) contained 0.5 µM of each primer and 1 µL of template DNA, with a no-template control. The two reads were trimmed and merged as described above, giving a 519-bp contig (412-bp overlap without mismatch) that was 100% identical (519/519) to the rrnS region of complete mitogenomes of both *Anisakis simplex* sensu stricto (GenBank OQ354213.1, NC_007934.1) and *Anisakis pegreffii* (NC_034329.1) and to *A. pegreffii* rrnS haplotypes (MT312511.1), and 99% identical to the remaining *A. simplex* (sensu lato) mitogenomes. Because the rrnS segment does not discriminate between *A. simplex* sensu stricto and *A. pegreffii*, the nematodes are reported as *A. simplex* (sensu lato); the sequence and chromatograms are provided in Supplementary Data S1.

## 3. Discussion

In this juvenile common dolphin, the combination of pathognomonic rhomboid skin lesions, sanguineous body-cavity effusions, pulmonary congestion, haemorrhage and oedema with acute reactive lymphadenitis, and the detection of *E. rhusiopathiae* DNA in all 12 tissues examined, including cardiac blood, supports a diagnosis of systemic infection consistent with fatal septicaemic erysipelas with concurrent dermatological involvement. Because the organism was not cultured and its identity within the lesions was not confirmed by immunohistochemistry or in situ hybridisation, although Gram staining demonstrated abundant Gram-positive bacteria throughout the necrotic epidermis and dermis of the skin lesions (Figure 3I,J), *E. rhusiopathiae* septicaemia is regarded here as the probable, rather than the definitively demonstrated, cause of death. The organism was detected in cardiac blood and in every parenchymal organ and lymph node examined, a distribution that matches the pattern described in septicaemic delphinids from England and Wales, in which *E. rhusiopathiae* was most consistently isolated from liver, kidney and brain [10], and in the rough-toothed dolphin stranded in Korea [15]. In skeletal muscle collected from an unaffected region, no *Erysipelothrix* sequence could be resolved from the broad-range 16S rRNA amplicon, but the genus- and species-directed assays were positive with a weaker band than in the other tissues; this pattern is most consistent with a lower bacterial load in muscle that fell below the dominance threshold of the broad-range assay while remaining detectable by targeted PCR, as expected in terminal bacteraemia. Several observations argue against post-mortem overgrowth or carry-over contamination as an explanation for the multi-organ positivity. No *E. rhusiopathiae* culture, isolate or positive-control DNA had ever been handled in the laboratory in which the extractions and PCRs were performed, so that the only possible source of *Erysipelothrix* DNA was the carcass itself; each tissue was sampled with fresh instruments and extracted separately; the no-template controls and, in the confirmatory run performed on re-extracted DNA from all 12 tissues, the extraction blank, an uninfected-dolphin tissue extract and *E. coli* DNA remained negative; the same organism was recovered from anatomically separate compartments, including cardiac blood; the broad-range assay, which would have revealed a dominant overgrowth flora, returned an *Erysipelothrix* sequence as the dominant amplicon in 11 of 12 tissues; and the molecular distribution matched the gross and histological lesions. Broad-range 16S rRNA sequencing cannot by itself distinguish *E. rhusiopathiae* from the closely related and less pathogenic *E. tonsillarum*, whose 16S rRNA sequences are nearly identical, and the same limitation applies to the genus-directed MO101/MO102 amplicon; the species assignment therefore rests on the sequence of the ER1 locus, which separated the present sequence from *E. tonsillarum*, *E. piscisicarius* and the recently described marine-mammal- and seabird-associated species *E. enhydrae* and *E. amsterdamensis*, as applied in previous cetacean and avian cases in which PCR complemented or replaced culture [15,16,30]. Sequencing of this locus showed that the strain detected here belongs to the main *E. rhusiopathiae* lineage. It was most similar to the isolate from the rough-toothed dolphin stranded on the southern coast of Korea in 2018 [15], with which it shared one of its three substitutions; however, the genome-wide survey showed that this substitution is a minor allele that also occurs in unrelated isolates from pigs on three continents (Supplementary Table S4), and the other two substitutions were unique to the present case. One shared nucleotide at a single 356-bp locus in two animals examined eight years apart therefore does not demonstrate a shared or regional lineage; at most it provides a hypothesis that could be tested by whole-genome or multilocus comparison should an isolate from a Korean cetacean become available. That the dolphin allele is nevertheless distinct from all 230 public genomes most probably reflects the composition of the public collection, which is dominated by porcine isolates and contains almost no *E. rhusiopathiae* genomes from marine mammals, rather than a unique lineage; whether cetacean strains in the Northwest Pacific form a distinct group, as *E. rhusiopathiae* subsp. *ohloneorum* does in sea otters, can be resolved only by isolating and sequencing further strains.

Table 1 places the case among the published reports of *E. rhusiopathiae* infection in free-ranging cetaceans. Systemic infection, with or without rhomboid skin lesions, has been described in bottlenose dolphins, harbour porpoises, an Atlantic spotted dolphin, a rough-toothed dolphin and a common dolphin from the North Atlantic, the Mediterranean, the eastern Pacific, the South Atlantic and the Korean south coast [7,8,10,11,12,13,14,15], and the authors of the recent clusters in the Netherlands and California have raised the possibility that the disease is emerging in response to environmental change [12,13]. All of these reports, with a single exception limited to PCR of skin [11], established the diagnosis from a cultured isolate, and strain characterisation, where attempted, likewise relied on cultured isolates, by whole-genome sequencing [13,15] or multilocus sequence analysis [14]. The present case differs in three respects. First, to our knowledge it is the only report in which the disease was imaged before dissection, so that the pulmonary and peritoneal changes and the gastric foreign body were documented in situ and could be correlated with necropsy and histology. Second, because the carcass had been frozen, the diagnosis was established without culture through a tiered molecular approach that combined broad-range 16S rRNA sequencing, genus- and species-specific PCR and sequencing of the species-specific rRNA-operon locus in 12 tissues; this workflow resolved the *E. rhusiopathiae*/*E. tonsillarum* ambiguity inherent to 16S rRNA data, because the species assignment was based on sequencing of a species-discriminating locus in every tissue rather than on 16S rRNA data or on the species-directed PCR alone, and it revealed the graded tissue distribution discussed above. Third, it provides a complete tissue-by-tissue molecular dataset (Table 2) in which the sequence obtained directly from tissue was compared with the genome of the only other *E. rhusiopathiae* isolate from a Korean cetacean and with all 230 *E. rhusiopathiae* genome assemblies that cover the locus, a comparison that defines what a single-locus sequence can and cannot say about strain relatedness. These elements, rather than the geographic record alone, are what we consider the contribution of the case.

Rhomboid (“diamond-skin”) lesions are considered pathognomonic for erysipelas in both swine and cetaceans [1,4,5]. In pigs, the lesions arise from bacterial localisation in the dermal microvasculature with endothelial injury, microthrombosis and ischaemic necrosis of the overlying skin, aided by virulence factors such as neuraminidase, hyaluronidase and the capsule, so that the geometric outline of each plaque reflects the territory of the affected vessels [1,2]. In the present case, histology of the lesions showed full-thickness coagulative necrosis of the epidermis and dermal papillae with a neutrophilic-to-mixed infiltrate and necrotising septal panniculitis of the superficial blubber, whereas the underlying skeletal muscle was spared in the sections examined. Bacterial aggregates were seen only within subepidermal clefts and on the ulcerated surface, where they were accompanied by a dense neutrophilic band; such surface aggregates in a stranded carcass may represent *E. rhusiopathiae* but may equally represent post-mortem or secondary colonisation, and the organism was not identified morphologically within the intact dermis in the haematoxylin and eosin sections. Gram staining, however, revealed large numbers of Gram-positive bacteria throughout the necrotic epidermis and dermis of the lesion, extending well beyond the ulcerated surface (Figure 3I,J); this distribution argues against surface colonisation alone and corresponds to the dermal localisation of *E. rhusiopathiae* in erysipelas, although the Gram stain cannot by itself assign the organisms to species. Freeze–thaw artefact materially limited the assessment of the dermal microvasculature: ice-crystal clefts disrupted vessel walls, nuclear detail of endothelial cells was lost, and post-mortem intravascular clotting could not be separated from ante-mortem fibrin thrombi, so that the endothelial injury, fibrinoid necrosis and microthrombosis that are presumed to underlie the geometric outline of the lesions [1,2] could be neither confirmed nor excluded. The histological diagnosis of the skin lesions therefore rests on the pattern of full-thickness coagulative necrosis with a sharply demarcated geometric outline rather than on direct demonstration of vasculitis. The lesion morphology nevertheless corresponds closely to the descriptions in captive dolphins [5] and in free-ranging bottlenose dolphins from the Mediterranean and Brazil [7,11,14]. Notably, the rough-toothed dolphin from Korean waters died of systemic infection without rhomboid skin lesions [15], underscoring that the dermatological and septicaemic forms are not mutually exclusive and that the absence of rhomboid plaques does not exclude erysipelas in a stranded cetacean.

The systemic lesions were those of an acute bacterial septicaemia rather than of an organ-specific infection. Pulmonary congestion with alveolar haemorrhage and proteinaceous oedema, without a bronchopneumonic exudate, together with sanguineous pleural and peritoneal effusions, is consistent with the increased vascular permeability that characterises acute erysipelas in dolphins [5,7,8,9], and the enlargement of the cervical lymph nodes together with sinus oedema, histiocytosis and neutrophil influx in both peripheral and visceral nodes reflects a generalised reactive response to bacteraemia. The interstitial nephritis and the neutrophil-rich splenic red pulp are similarly compatible with haematogenous dissemination; in the Korean rough-toothed dolphin, *E. rhusiopathiae* was likewise isolated from blood, kidney and pleural fluid, and generalised lymphadenomegaly with pulmonary congestion was among the principal gross findings [15]. Bacterial emboli were not seen in the parenchymal organs in the haematoxylin and eosin sections, in contrast to several previous reports [8,10,12]; because Gram staining was applied only to the skin lesions, where it revealed far more organisms than were apparent on haematoxylin and eosin, the absence of visible bacteria in the other organs should not be taken as evidence of a low tissue load, and the distribution of the organism rests on the molecular findings (Table 2). None of these lesions is specific, however, and in the absence of the skin plaques the differential diagnosis would have included other bacterial septicaemias of cetaceans, which is why molecular confirmation across multiple tissues was essential.

To our knowledge, this is the first report in which cetacean erysipelas has been imaged by PMCT, and whole-body PMCT performed before necropsy added information that was either not accessible or would have been altered by dissection [23,33,34,35]. PMCT did not, and could not, establish the aetiological diagnosis: the imaging pattern documented here, a gravity-dependent gradient of pulmonary opacification with fluid in the central airways and a dependent peritoneal effusion in the absence of decomposition gas, is not specific for erysipelas and would be expected in other septicaemias and in several non-infectious causes of pulmonary oedema, but it provides a reference for the in situ appearance of septicaemic pulmonary oedema and haemorrhage in a frozen cetacean carcass against which future cases can be compared. These changes were documented in situ and correlated with the wet, fluid-exuding lungs found at necropsy; the peritoneal effusion and the metallic foreign body in the fundic stomach were identified before the abdomen was opened, allowing targeted retrieval of the hook and sampling of the adjacent gastric wall; and the absence of intravascular, subcutaneous or intra-organ gas provided an objective indication that decomposition was minimal and that the imaging and histological findings were interpretable. These contributions are in line with our previous experience with virtopsy in a long-term monitored Indo-Pacific bottlenose dolphin, in spotted seals with vessel-strike trauma and in finless porpoises with vertebral deformities [20,21,23]. The limitations of non-contrast PMCT of a frozen carcass should also be acknowledged: individual lymph nodes could not be delineated or measured, the sanguineous pleural fluid found at necropsy could not be separated from the consolidated dependent lung on the images, and the pulmonary pattern cannot by itself separate oedema, haemorrhage and pneumonia, which required histology. PMCT therefore complements rather than replaces conventional necropsy in stranded cetaceans, but it is particularly valuable when, as here, the carcass must be stored frozen before examination.

Two incidental findings deserve comment. First, a corroded fishing hook was embedded in a markedly thickened, mucus-covered fundic stomach wall, gross changes suggestive of a chronic foreign-body gastritis that indicate a fisheries interaction well before the terminal illness; the hook site was not included in the histological sections, so the gastritis was not confirmed microscopically. Common dolphins are the most frequently bycaught delphinid in Korean waters [18], and ingestion of hooked bait or discarded gear is a recognised route of interaction. Second, numerous nematodes were embedded in the forestomach wall; mitochondrial rrnS sequencing identified them as members of the *A. simplex* complex (*A. simplex* sensu lato), the anisakid group that is widespread in fish and cetaceans of Korean waters, where *A. pegreffii* has been recorded as the adult stage in finless porpoises [36]. *E. rhusiopathiae* is carried in the mucous slime of marine fish and can be recovered from a substantial proportion of seafood samples [1,6], and infection in cetaceans is thought to follow ingestion of contaminated fish, with the gastric or oral mucosa as the presumed portal of entry [4,5]. Mucosal injury caused by a retained foreign body or by parasite attachment could plausibly facilitate bacterial invasion, although a causal link cannot be established from a single case.

Although *E. rhusiopathiae* septicaemia has already been recorded in a common dolphin in the North Atlantic, in which no skin lesions were observed [10], this is, to our knowledge, the first confirmed case in the species from the Northwest Pacific, the first in which the rhomboid dermatological form has been documented in *D. delphis*, and the second confirmed case of cetacean erysipelas in Korean waters after the rough-toothed dolphin from Yeosu in 2018 [15]. Common dolphins have until recently been rarely available for post-mortem examination in the region: only 18 of 557 cetacean carcasses recorded around Jeju Island between 2016 and July 2026 belonged to this species, compared with the overwhelming predominance of narrow-ridged finless porpoises, so that the pathogen exposure of common dolphins in these waters is essentially undocumented [18,19]. Notably, 11 of these 18 carcasses were recorded in the 20 months preceding this report, all in the winter half-year, together with sightings of large pods off Jeju (Supplementary Table S3). The recorded proportion of common dolphins among carcasses has therefore increased; whether this reflects a change in the winter distribution of the species around Jeju or changes in surveillance effort, carcass detection and reporting cannot be determined from stranding data alone, and we do not interpret it as a population trend, but it suggests that further cases in this species may be encountered by the regional stranding network. The present case, together with the 2018 rough-toothed dolphin [15], shows that *E. rhusiopathiae* infection occurs in delphinids of the southern Korean coast and Jeju and that erysipelas should be considered in the differential diagnosis of sudden death in stranded odontocetes, whether or not skin lesions are present. The finding also has a direct occupational implication: *E. rhusiopathiae* causes erysipeloid and, rarely, endocarditis in people who handle infected animals or contaminated fish [2], and stranding-response personnel who examine carcasses with geometric skin lesions should wear gloves and protective clothing and avoid cutaneous injuries.

This report has several limitations. Culture was not attempted because the carcass had been stored frozen, so no isolate is available for serotyping, antimicrobial susceptibility testing or whole-genome comparison with the Korean rough-toothed dolphin isolate [15]; although *E. rhusiopathiae* can be recovered from frozen tissues, the prolonged storage and repeated handling of this carcass were judged to make a negative culture uninterpretable. The aetiological diagnosis therefore rests on species-directed PCR and amplicon sequencing in 12 tissues, supported by broad-range 16S rRNA sequencing, no reference strain was available as a positive control, the identity of the amplicons being established by sequencing instead, and an extraction blank was not included in the original extractions but was included, together with negative-tissue and *E. coli* controls, in the confirmatory run on re-extracted DNA from all 12 tissues. The phylogenetic placement of the detected strain is limited to a single 356-bp locus that resolves species but not strain relationships, and the comparison with the 2018 Korean isolate should be read in that light. Freeze–thaw artefact reduced the quality of the histological sections and precluded assessment of dermal vascular lesions, and although Gram staining localised abundant Gram-positive bacteria throughout the skin lesions, their identity as *E. rhusiopathiae* was not confirmed in situ by immunohistochemistry or in situ hybridisation, and Gram staining was not extended to the parenchymal organs, so the histological localisation of the organism outside the skin remains undocumented. Finally, PMCT was performed without contrast on a frozen carcass and was read qualitatively. Despite these constraints, the convergence of gross, histological, imaging and molecular findings provides a coherent picture of disseminated erysipelas in this animal.

## 4. Conclusions

This case of systemic *E. rhusiopathiae* infection with classical rhomboid dermatitis in a free-ranging short-beaked common dolphin from Korean waters, in which septicaemic erysipelas was the probable cause of death, adds three elements to the literature on cetacean erysipelas: a PMCT description of the disease, with imaging findings that were correlated with necropsy and histopathology; a culture-independent, tiered molecular workflow that established the species identity by amplicon sequencing in all 12 tissues of a frozen carcass and excluded *E. tonsillarum* and other *Erysipelothrix* species; and a tissue-by-tissue molecular dataset with a direct-from-tissue sequence that, when compared with all available *E. rhusiopathiae* genomes, is distinct from every genome examined but does not by itself establish its relationship to the earlier Korean cetacean isolate. Erysipelas should be included in the differential diagnosis of sudden death and of geometric skin lesions in delphinids of the region, molecular methods that discriminate *E. rhusiopathiae* from other *Erysipelothrix* species should be applied when culture is not feasible, and stranding-response teams should apply appropriate biosafety measures because of the zoonotic potential of the organism.

## Figures and Tables

**Table 1:** Published reports of *Erysipelothrix rhusiopathiae* infection in free-ranging delphinids and porpoises identified in this review, and the present case.

Species (n)	Location, Year	Presentation	Diagnostic Basis	Strain or Sequence Data	Ref.
*Tursiops truncatus* (1)	Spain (Mediterranean), 2010	Rhomboid skin lesions; septicaemia	Culture; histology	None	[7]
*Stenella frontalis* (1); *T. truncatus* (1)	Spain (Canary Is.), 2010, 2012	Septicaemia; no skin lesions	Culture; IHC; EM	None	[8]
*T. truncatus* (3); *Phocoena phocoena* (3); *Delphinus delphis* (1)	England and Wales, 1990–2019	Septicaemia; no skin lesions	Culture; biochemical ID; histology	None	[10]
*T. truncatus* (1)	Brazil, 2020	Rhomboid skin lesions; dermatitis with vasculitis	PCR of skin (*Erysipelothrix* sp.); histology	None	[11]
*T. truncatus* (3)	USA (California), 2022	Septicaemia (cluster)	Culture; MALDI-TOF; histology	None	[12]
*P. phocoena* (mass mortality; 16/21 culture-positive)	Netherlands, 2021	Mass mortality; carcasses decomposed	Culture (liver)	WGS of 18 isolates (two clonal lineages)	[13]
*T. truncatus* (1)	Spain (Catalonia), 2022	Rhomboid skin lesions; meningeal vasculitis	Culture; MLSA	MLSA of isolate	[14]
*Steno bredanensis* (1)	Republic of Korea (Yeosu), 2018	Septicaemia; no skin lesions	Culture; histology	WGS of isolate KC-Sb-R1	[15]
*D. delphis* (1)	Republic of Korea (Jeju), 2026	Rhomboid skin lesions; septicaemia; gastric foreign body	PMCT; histology; culture-independent PCR and amplicon sequencing in 12 tissues (Table 2)	Direct-from-tissue 16S rRNA and ER1 sequences; comparison with 230 genomes	This study

EM, electron microscopy; ID, identification; IHC, immunohistochemistry; MLSA, multilocus sequence analysis; PMCT, post-mortem computed tomography; WGS, whole-genome sequencing. Cases in captive cetaceans and in other cetacean taxa, reviewed in [4,5,9,15], are not listed. None of the previous reports included post-mortem imaging. The seven animals in [10] showed septicaemia in all cases without rhomboid or ulcerative skin lesions; PCR and sequencing were not performed.

## Data Availability

Nucleotide sequences generated in this study have been deposited in GenBank under accession numbers PZ953691 and PZ953692 (*Erysipelothrix rhusiopathiae* 16S rRNA gene, cardiac blood and lung), PZ956008 (MO101/MO102 amplicon, skin lesion), PZ983794 (ER1F/ER1R amplicon, skin lesion), and PZ946319 and PZ946320 (*Delphinus delphis* cytochrome b and control region). All raw Sanger chromatograms (including the Anisakis rrnS reads, which were not deposited), assembled contigs, alignments, tree files and analysis scripts are provided in the Supplementary Materials (Data S1), and the uncropped gel images are shown in Supplementary Figures S2 and S3. The PMCT image series is available from the corresponding authors on reasonable request. Stranding records for Jeju Island were provided by the Whale–Marine Life Conservation Research Center, Gidang Institute of Marine Science, Jeju National University, and are available from that centre on request.

## Supplementary Materials

The following supporting information can be downloaded at: https://www.mdpi.com/article/10.3390/vetsci13101125/s1, Table S1: GenBank sequences used as references for the phylogenetic analysis of the ER1F/ER1R amplicon, with the number of mismatches to the ER1F and ER1R primers; Table S2: BLASTn results for the mitochondrial cytochrome b and control-region (D-loop) sequences used to confirm the species identity of the stranded dolphin; Table S3: Common dolphin (*Delphinus delphis*) carcasses recorded around Jeju Island, January 2024–July 2026; Table S4: Alleles at the three variable positions of the ER1 locus in 230 *Erysipelothrix rhusiopathiae* genome assemblies (summary; full list in Table S4.xlsx); Figure S1: Maximum-likelihood phylogeny of the ER1F/ER1R amplicon of *Erysipelothrix rhusiopathiae* detected in the dolphin; Figure S2: Uncropped agarose gel electrophoresis of the original PCR run with the *Erysipelothrix* genus-directed (MO101/MO102) and *E. rhusiopathiae*-directed (ER1F/ER1R) primers on DNA extracts of 12 tissues of the dolphin; Figure S3: Uncropped agarose gel electrophoresis of the confirmatory PCR run performed during revision on DNA re-extracted from all 12 tissues, with extraction blank, uninfected-dolphin tissue, *Escherichia coli* DH5α and no-template controls; Data S1. Raw Sanger chromatograms (.ab1) of all Erysipelothrix, mitochondrial and *Anisakis* reads, assembled contigs, ER1 alignment and tree files, and analysis scripts (separate archive).

## Abbreviations

The following abbreviations are used in this manuscript:

ATCC	American Type Culture Collection
BLAST	Basic Local Alignment Search Tool
bp	base pair(s)
CT	Computed Tomography
D-loop	mitochondrial control region (Displacement loop)
EM	Electron Microscopy
FIRA	Korea Fisheries Resources Agency
H&E	Haematoxylin and Eosin
HU	Hounsfield Unit
IHC	Immunohistochemistry
MLSA	Multilocus Sequence Analysis
NCBI	National Center for Biotechnology Information
PCR	Polymerase Chain Reaction
PMCT	Post-mortem Computed Tomography
rRNA	Ribosomal RNA
WGS	Whole-genome Sequencing

## References

[1] Wang Q., Chang B.J., Riley T.V. (2010). *Erysipelothrix* *rhusiopathiae*. Vet. Microbiol..

[2] Brooke C.J., Riley T.V. (1999). *Erysipelothrix rhusiopathiae*: Bacteriology, epidemiology and clinical manifestations of an occupational pathogen. J. Med. Microbiol..

[3] Shimoji Y. (2000). Pathogenicity of *Erysipelothrix rhusiopathiae*: Virulence factors and protective immunity. Microbes Infect..

[4] Dunn J.L., Buck J.D., Robeck T.R., Dierauf L.A., Gulland F.M.D. (2001). Bacterial diseases of cetaceans and pinnipeds. CRC Handbook of Marine Mammal Medicine.

[5] Geraci J.R., Sauer R.M., Medway W. (1966). Erysipelas in dolphins. Am. J. Vet. Res..

[6] Fidalgo S.G., Wang Q., Riley T.V. (2000). Comparison of methods for detection of *Erysipelothrix* spp. and their distribution in some Australasian seafoods. Appl. Environ. Microbiol..

[7] Melero M., Rubio-Guerri C., Crespo J.L., Arbelo M., Vela A.I., García-Párraga D., Sierra E., Domínguez L., Sánchez-Vizcaíno J.M. (2011). First case of erysipelas in a free-ranging bottlenose dolphin (*Tursiops truncatus*) stranded in the Mediterranean Sea. Dis. Aquat. Org..

[8] Díaz-Delgado J., Arbelo M., Sierra E., Vela A., Domínguez M., Paz Y., Andrada M., Domínguez L., Fernández A. (2015). Fatal *Erysipelothrix rhusiopathiae* septicemia in two Atlantic dolphins (*Stenella frontalis* and *Tursiops truncatus*). Dis. Aquat. Org..

[9] Kinsel M.J., Boehm J.R., Harris B., Murnane R.D. (1997). Fatal *Erysipelothrix rhusiopathiae* septicemia in a captive Pacific white-sided dolphin (*Lagenorhynchus obliquidens*). J. Zoo Wildl. Med..

[10] Ceccolini M.E., Wessels M., Macgregor S.K., Deaville R., Perkins M., Jepson P.D., John S.K., Guthrie A. (2021). Systemic *Erysipelothrix rhusiopathiae* in seven free-ranging delphinids stranded in England and Wales. Dis. Aquat. Org..

[11] Sacristán C., Ewbank A.C., Sánchez-Sarmiento A.M., Duarte-Benvenuto A., Borges J.C.G., Rebelo V.A., Díaz-Delgado J., Keid L.B., Catão-Dias J.L. (2022). Erysipelas in a stranded common bottlenose dolphin: First report in a South American odontocete. Braz. J. Microbiol..

[12] Danil K., Colegrove K.M., Delaney M.A., Mena A., Stedman N., Wurster E. (2023). Systemic erysipelas outbreak among free-ranging bottlenose dolphins, San Diego, California, USA, 2022. Emerg. Infect. Dis..

[13] IJsseldijk L.L., Begeman L., Duim B., Oude Munnink B.B., Gröne A., Klijnstra M.D., Kik M.J.L., Lakemeyer J., ten Doeschate M., Leopold M.F. (2023). Harbor porpoise deaths associated with *Erysipelothrix rhusiopathiae*, the Netherlands, 2021. Emerg. Infect. Dis..

[14] Martino L., Serrano B., Alomar J., Pérez L., Aragon V., Cobos À., Abarca M.L., Yazdi Z., Soto E., Domingo M. (2024). Erysipelas with preferential brain and skin involvement in a Mediterranean bottlenose dolphin *Tursiops truncatus*. Dis. Aquat. Org..

[15] Lee K., Park S.Y., Seo H.W., Cho Y., Choi S.-G., Seo S., Han W., Lee N.-K., Kwon H., Han J.E. (2022). Pathological and genomic findings of *Erysipelothrix rhusiopathiae* isolated from a free-ranging rough-toothed dolphin *Steno bredanensis* (Cetacea: Delphinidae) stranded in Korea. Front. Vet. Sci..

[16] Boerner L., Nevis K.R., Hinckley L.S., Weber E.S., Frasca S. (2004). *Erysipelothrix* septicemia in a little blue penguin (*Eudyptula minor*). J. Vet. Diagn. Investig..

[17] IJsseldijk L.L., Brownlow A.C., Mazzariol S. (2019). Best Practice on Cetacean Post Mortem Investigation and Tissue Sampling.

[18] Yoo J.-T., Park K.J., Lee K., Lee D. (2023). A first study on distribution characteristics of common dolphin in Korean waters: A study using data collected during the past 20 years. J. Mar. Sci. Eng..

[19] Song K.-J. (2016). Cetacean strandings in Korean waters. Pac. Sci..

[20] Park J.-H., Lee K.L., Lee Y.M., Choi J.Y., Heo Y.R., Oh S.M., Lee D., Kim S., Lee H.W., Poon C.T.C. (2026). From traumatic oral fibroma to fatal pneumonia: A multidisciplinary postmortem investigation in a long-term monitored Indo-Pacific bottlenose dolphin (*Tursiops aduncus*). BMC Zool..

[21] Park J.-H., Choi H.S., Noh D., Choi S., Seok S.H., Kim S.W., Yuen A.H.L. (2026). Anthropogenic vessel strike as a threat to spotted seals (*Phoca largha*) in Korean waters: A multimodal forensic investigation. Animals.

[22] Kim S.W., Yuen A.H.L., Kim H.W., Lee S., Lee S.B., Lee Y.M., Jung W.J., Poon C.T.C., Park D., Kim S. (2025). Digital removal of dermal denticle layer using geometric AI from 3D CT scans of shark craniofacial structures enhances anatomical precision. Sci. Rep..

[23] Yuen A.H.L., Kim S.W., Lee K., Lee Y.M., Lee S.B., Kim M.J., Poon C.T.C., Jung W.J., Jo S.J., Hwang M.H. (2024). First report of kyphoscoliosis in the narrow-ridged finless porpoises (*Neophocaena asiaeorientalis*): Findings from congenital and degenerative cases comparison using post-mortem computed tomography. Vet. Med. Sci..

[24] Cassens I., Vicario S., Waddell V.G., Balchowsky H., Van Belle D., Ding W., Fan C., Mohan R.S.L., Simões-Lopes P.C., Bastida R. (2000). Independent adaptation to riverine habitats allowed survival of ancient cetacean lineages. Proc. Natl. Acad. Sci. USA.

[25] Baker C.S., Perry A., Bannister J.L., Weinrich M.T., Abernethy R.B., Calambokidis J., Lien J., Lambertsen R.H., Ramírez J.U., Vasquez O. (1993). Abundant mitochondrial DNA variation and world-wide population structure in humpback whales. Proc. Natl. Acad. Sci. USA.

[26] Dalebout M.L., van Helden A., van Waerebeek K., Baker C.S. (1998). Molecular genetic identification of southern hemisphere beaked whales (Cetacea: Ziphiidae). Mol. Ecol..

[27] Ross H.A., Lento G.M., Dalebout M.L., Goode M., Ewing G., McLaren P., Rodrigo A.G., Lavery S., Baker C.S. (2003). DNA Surveillance: Web-based molecular identification of whales, dolphins, and porpoises. J. Hered..

[28] Möller L.M., Beheregaray L.B. (2001). Coastal bottlenose dolphins from southeastern Australia are *Tursiops aduncus* according to sequences of the mitochondrial DNA control region. Mar. Mamm. Sci..

[29] Relman D.A., Loutit J.S., Schmidt T.M., Falkow S., Tompkins L.S. (1990). The agent of bacillary angiomatosis: An approach to the identification of uncultured pathogens. N. Engl. J. Med..

[30] Makino S., Okada Y., Maruyama T., Ishikawa K., Takahashi T., Nakamura M., Ezaki T., Morita H. (1994). Direct and rapid detection of *Erysipelothrix rhusiopathiae* DNA in animals by PCR. J. Clin. Microbiol..

[31] Takeshi K., Makino S., Ikeda T., Takada N., Nakashiro A., Nakanishi K., Oguma K., Katoh Y., Sunagawa H., Ohyama T. (1999). Direct and rapid detection by PCR of *Erysipelothrix* sp. DNAs prepared from bacterial strains and animal tissues. J. Clin. Microbiol..

[32] D’Amelio S., Barros N.B., Ingrosso S., Fauquier D.A., Russo R., Paggi L. (2007). Genetic characterization of members of the genus *Contracaecum* (Nematoda: Anisakidae) from fish-eating birds from west-central Florida, USA, with evidence of new species. Parasitology.

[33] Yuen A.H.L., Kim S.W., Lee S.B., Lee S., Lee Y.R., Kim S.M., Poon C.T.C., Kwon J., Jung W.J., Giri S.S. (2022). Radiological investigation of gas embolism in the East Asian finless porpoise (*Neophocaena asiaeorientalis sunameri*). Front. Mar. Sci..

[34] Yuen A.H.L., Lee S.B., Kim S.W., Lee Y.M., Kim D.-G., Poon C.T.C., Seo J.-P., Baeck G.W., Kim B.Y., Park S.C. (2024). Fatal upper aerodigestive tract obstruction in an East Asian finless porpoise (*Neophocaena asiaeorientalis sunameri*): Findings in post-mortem computed tomography. Forensic Sci. Med. Pathol..

[35] Lee S.B., Yuen A.H.L., Kim S., Jung W.J., Kim D.-G., Kim S.W., Lee Y.M., Park D., Cho H.S., Poon C.T.C. (2024). Ingestion of fishing gear and *Anisakis* sp. infection in a beached Indo-Pacific finless porpoise (*Neophocaena phocaenoides*) in the Jeju Island, Republic of Korea: Findings from post-mortem computed tomography and necropsy. BMC Vet. Res..

[36] Kim S., Jeon J.Y., Lee K., Lee H., Lee H., Lee S.B., Kim S.W., Park S.C., Choe S., Youn H. (2024). Genetic analyses of *Anisakis pegreffii* (Nematoda: Anisakidae) from the East Asian finless porpoise *Neophocaena asiaeorientalis* sunameri (Cetacea: Phocoenidae) in Korean waters. Parasitol. Res..

