# Supplementary material for: Systemic Erysipelothrix rhusiopathiae Infection with Rhomboid Skin Lesions in a Free-Ranging Short-Beaked Common Dolphin (Delphinus delphis) Stranded in Jeju, Republic of Korea: Post-Mortem Computed Tomography, Pathology and Multi-Tissue Molecular Detection
Source: Vet Sci. 2026 Oct 21;13(10):1125. doi: 10.3390/vetsci13101125 (PMC13709876; doi:10.3390/vetsci13101125)
Supplement: Supplementary file 1 [file vetsci-13-01125-s001.zip › vetsci-4584257-supplementary/Supplementary files/Supplementary_Data_R2_final.pdf]

Supplementary Data

Systemic *Erysipelothrix rhusiopathiae* Infection with Rhomboid Skin Lesions in a Free-Ranging Short-Beaked Common Dolphin (*Delphinus delphis*) Stranded in Jeju, Republic of Korea: Post-Mortem Computed Tomography, Pathology and Multi-Tissue Molecular Detection

Ji-Hyung Park, Yu Ri Heo, Young Jin Ryu, Ji Yeong Choi, Mohan Huang, Seyoung Lee, Jong-pil Seo, Adams Hei Long Yuen, Byung Yeop Kim, Seung Hyeok Seok and Sang Wha Kim

Contents

Data S1. Raw Sanger chromatograms (.ab1) of all Erysipelothrix, mitochondrial and *Anisakis* reads, assembled contigs, ER1 alignment and tree files, and analysis scripts (separate archive).

Data S1 (separate archive, SL26-0209A_Data_S1.zip). Raw Sanger chromatograms (.ab1) of all reads (16S rRNA 91E/13B, MO101/MO102 and ER1F/ER1R from 12 tissues; cytochrome b and control region; *Anisakis* rrnS MH3/MH4.5), assembled contigs (FASTA), per-tissue assembly report, the ER1 reference set, alignment (FASTA) and IQ-TREE output (treefile, .iqtree report), the ER1 genome-survey table and the Python/shell scripts used (Mott trimming, reference-guided merging, MAFFT/IQ-TREE pipeline).
