# Supplementary material for: Systemic Erysipelothrix rhusiopathiae Infection with Rhomboid Skin Lesions in a Free-Ranging Short-Beaked Common Dolphin (Delphinus delphis) Stranded in Jeju, Republic of Korea: Post-Mortem Computed Tomography, Pathology and Multi-Tissue Molecular Detection
Source: Vet Sci. 2026 Oct 21;13(10):1125. doi: 10.3390/vetsci13101125 (PMC13709876; doi:10.3390/vetsci13101125)
Supplement: Supplementary file 1 [file vetsci-13-01125-s001.zip › vetsci-4584257-supplementary/Supplementary files/Supplementary_Figures_R2_final.pdf]

# Supplementary Figures

## Systemic *Erysipelothrix rhusiopathiae* Infection with Rhomboid Skin Lesions in a Free-Ranging Short-Beaked Common Dolphin (*Delphinus delphis*) Stranded in Jeju, Republic of Korea: Post-Mortem Computed Tomography, Pathology and Multi-Tissue Molecular Detection

Ji-Hyung Park, Yu Ri Heo, Young Jin Ryu, Ji Yeong Choi, Mohan Huang, Seyoung Lee, Jong-pil Seo, Adams Hei Long Yuen, Byung Yeop Kim, Seung Hyeok Seok and Sang Wha Kim

**Contents**

Figure S1. Maximum-likelihood phylogeny of the ER1F/ER1R amplicon of *Erysipelothrix rhusiopathiae* detected in the dolphin.

Figure S2. Uncropped agarose gel electrophoresis of the original PCR run with the *Erysipelothrix* genus-directed (MO101/MO102) and *E. rhusiopathiae*-directed (ER1F/ER1R) primers on DNA extracts of 12 tissues of the dolphin.

Figure S3. Uncropped agarose gel electrophoresis of the confirmatory PCR run performed during revision on DNA re-extracted from all 12 tissues, with extraction blank, uninfected-dolphin tissue, *Escherichia coli* DH5$\alpha$ and no-template controls.

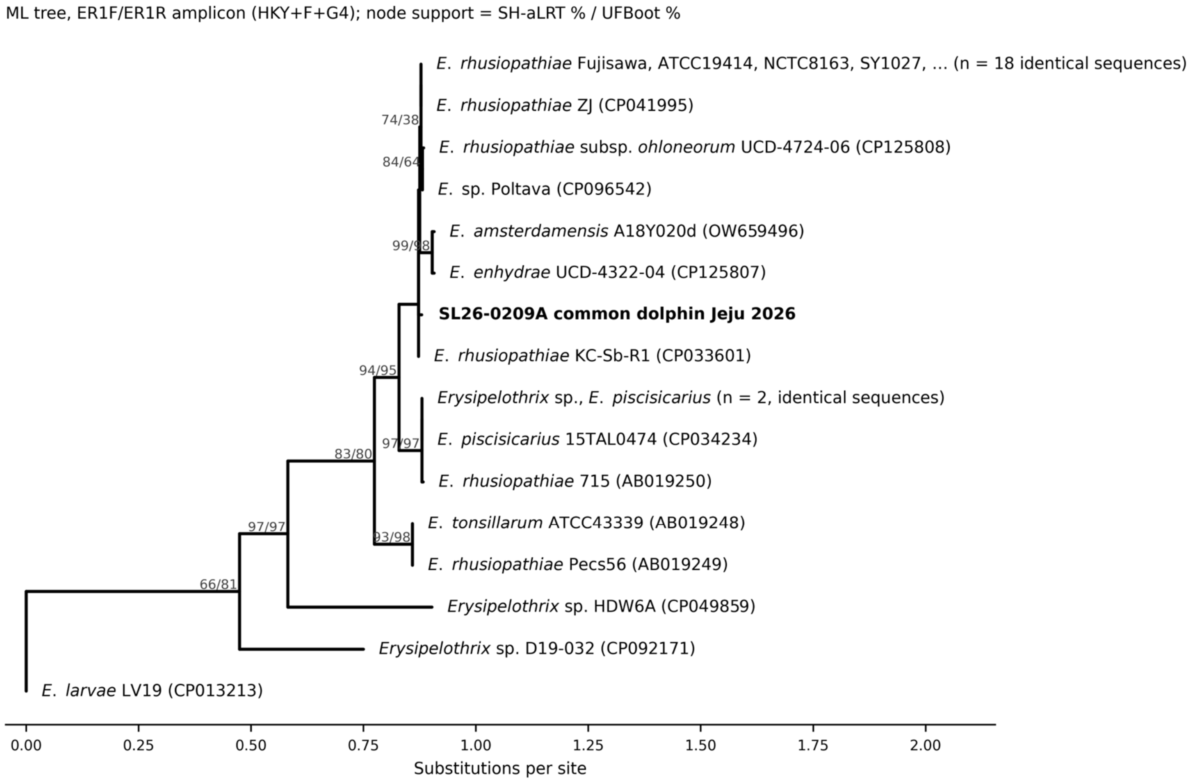


Figure S1. Maximum-likelihood tree of the ER1F/ER1R amplicon (non-coding region downstream of the 5S rRNA gene of the rRNA operon; 356 bp after removal of the primer-binding sites) of *Erysipelothrix rhusiopathiae* detected in the common dolphin SL26-0209A (bold; the sequence was identical in all 12 tissues examined, GenBank PZ983794) and of the homologous region of *Erysipelothrix* genomes and rRNA-operon records listed in Table S1. Sequences were aligned with MAFFT v7.505 (L-INS-i); primer-binding sites and columns with >50% gaps were removed (356 aligned positions), and the tree was inferred in IQ-TREE 2.0.7 under the best-fit model (HKY+F+G4, selected by ModelFinder/BIC) with 1,000 ultrafast bootstrap and 1,000 SH-aLRT replicates. Node labels give SH-aLRT/UFBoot support (%; shown when ≥70); identical reference sequences were collapsed into a single tip (n indicated). The tree is rooted on *E. larvae* LV19, the most divergent member of the genus. The SL26-0209A sequence differed from the *E. rhusiopathiae* type strain and 17 other genome-derived sequences at three positions (amplicon positions 262, 287 and 358) and from the Korean rough-toothed dolphin isolate KC-Sb-R1 (CP033601) at two (99.4% identity); identity to *E. piscisicarius* and *E. tonsillarum* was 90.7% and 86.5%, respectively. Scale bar, substitutions per site. Alignment and tree files are provided in Data S1.

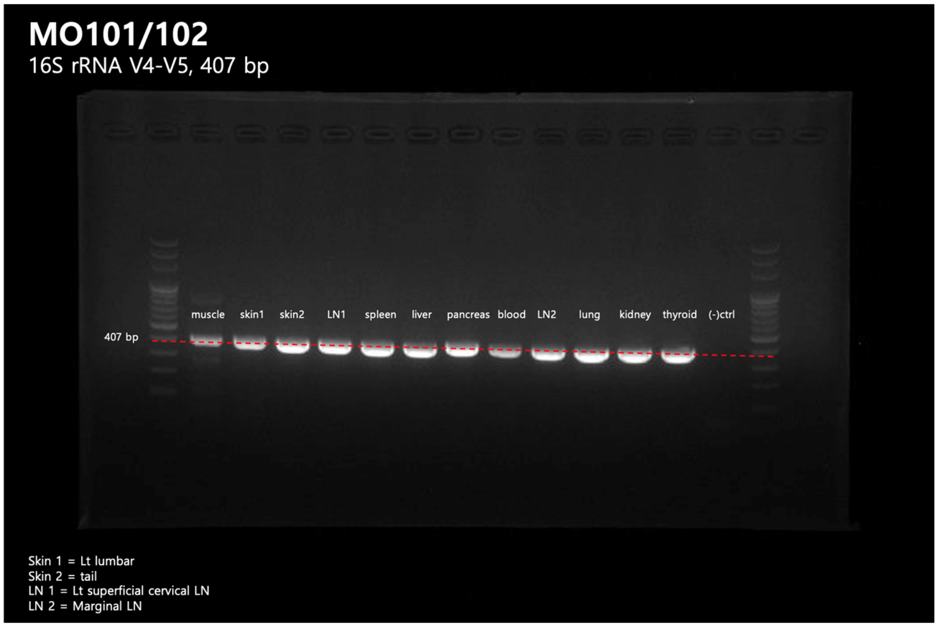


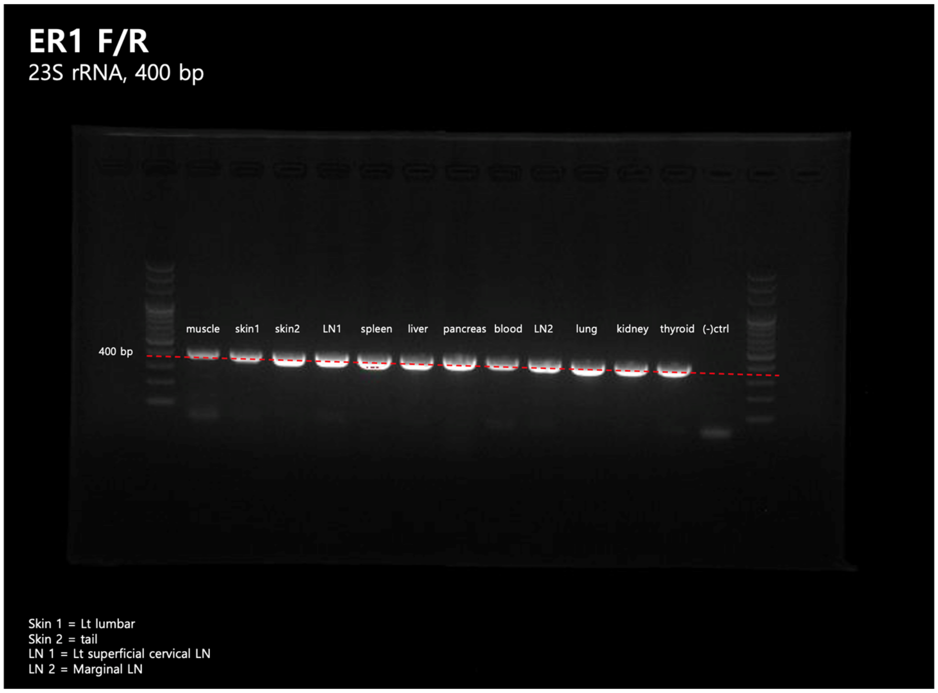


Figure S2. Uncropped agarose gel electrophoresis of the *Erysipelothrix* genus-directed (MO101/MO102; 16S rRNA gene, 407 bp; upper panel) and *E. rhusiopathiae*-directed (ER1F/ER1R; non-coding region downstream of the 5S rRNA gene, 400 bp; lower panel) PCR products from DNA extracts of 12 tissues of the common dolphin SL26-0209A (original run). Images are the complete, uncropped gels; the molecular-size ladder is loaded in the outer lanes and the lane map is printed on each image. Skin 1, left lumbar flank; skin 2, tail peduncle; LN1, left superficial cervical lymph node; LN2, marginal lymph node; (−) ctrl, no-template control.

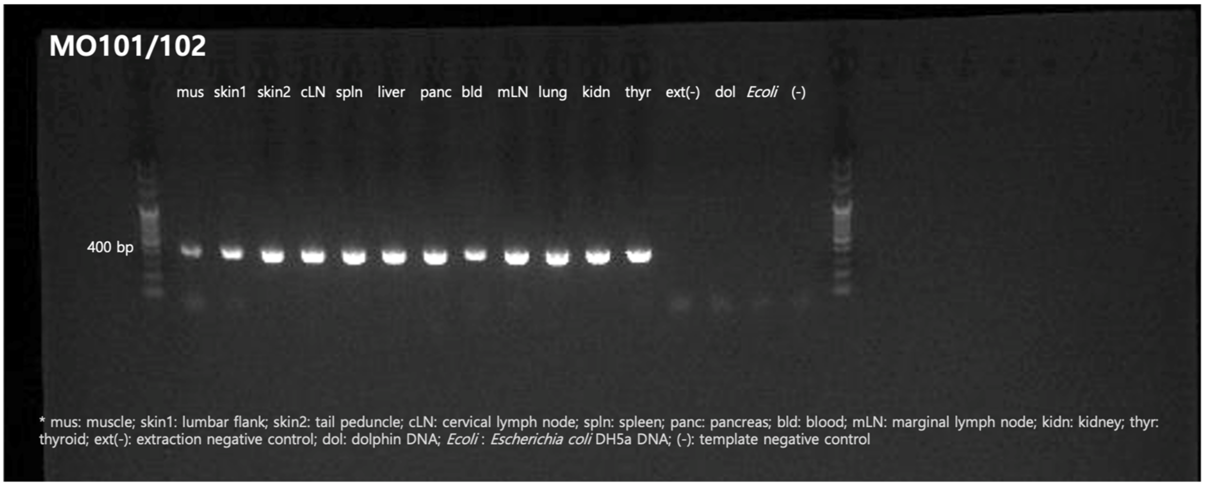


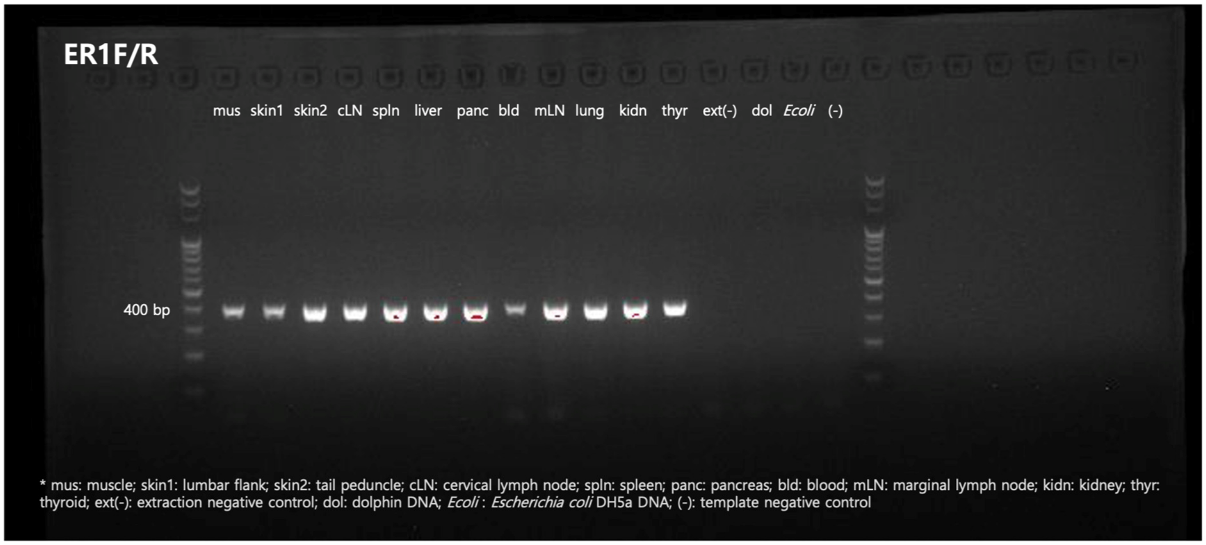


Figure S3. Uncropped agarose gel electrophoresis of the confirmatory PCR run performed during revision. DNA was re-extracted from all 12 tissues of the common dolphin SL26-0209A and amplified with the *Erysipelothrix* genus-directed primers MO101/MO102 (407 bp; upper gel) and the *E. rhusiopathiae*-directed primers ER1F/ER1R (400 bp; lower gel) alongside an extraction blank carried through the entire extraction procedure (ext(−)), DNA from a tissue of an unrelated, uninfected dolphin extracted with the same kit (dol), *Escherichia coli* DH5$\alpha$ DNA (*E. coli*) and a no-template control ((−)). Amplicons of the expected size were obtained from all 12 tissues with both assays, whereas all four controls remained negative. Molecular-size ladders are loaded in the outer lanes; images are complete and uncropped, and the lane map is printed on each image. mus, skeletal muscle; skin1, skin lesion 1; skin2, skin lesion 2; cLN, superficial cervical lymph node; spln, spleen; panc, pancreas; bld, cardiac blood; mLN, marginal lymph node; kidn, kidney; thyr, thyroid.
