# Supplementary material for: Systemic Erysipelothrix rhusiopathiae Infection with Rhomboid Skin Lesions in a Free-Ranging Short-Beaked Common Dolphin (Delphinus delphis) Stranded in Jeju, Republic of Korea: Post-Mortem Computed Tomography, Pathology and Multi-Tissue Molecular Detection
Source: Vet Sci. 2026 Oct 21;13(10):1125. doi: 10.3390/vetsci13101125 (PMC13709876; doi:10.3390/vetsci13101125)
Supplement: Supplementary file 1 [file vetsci-13-01125-s001.zip › vetsci-4584257-supplementary/Supplementary files/Supplementary_Tables_R2_final.pdf]

Supplementary Tables

Systemic *Erysipelothrix rhusiopathiae* Infection with Rhomboid Skin Lesions in a Free-Ranging Short-Beaked Common Dolphin (*Delphinus delphis*) Stranded in Jeju, Republic of Korea: Post-Mortem Computed Tomography, Pathology and Multi-Tissue Molecular Detection

Ji-Hyung Park, Yu Ri Heo, Young Jin Ryu, Ji Yeong Choi, Mohan Huang, Seyoung Lee, Jong-pil Seo, Adams Hei Long Yuen, Byung Yeop Kim, Seung Hyeok Seok and Sang Wha Kim

Contents

Table S1. GenBank sequences used as references for the phylogenetic analysis of the ER1F/ER1R amplicon, with the number of mismatches to the ER1F and ER1R primers.

Table S2. BLASTn results for the mitochondrial cytochrome b and control-region (D-loop) sequences used to confirm the species identity of the stranded dolphin.

Table S3. Common dolphin (*Delphinus delphis*) carcasses recorded around Jeju Island, January 2024–July 2026.

Table S4. Alleles at the three variable positions of the ER1 locus in 230 *Erysipelothrix rhusiopathiae* genome assemblies (summary; full list in Table S4.xlsx).

Table S1. GenBank sequences used as references for the phylogenetic analysis of the ER1F/ER1R amplicon (399–400 bp; non-coding region downstream of the 5S rRNA gene of the rRNA operon, positions 4119–4515 of AB019247) and number of mismatches of each sequence to the ER1F and ER1R primers (in silico re-assessment of assay specificity). Amplicon-equivalent regions were extracted in silico from complete genomes and rRNA-operon records with NCBI Primer-BLAST; for species not amplified by ER1F/ER1R, the homologous region was retrieved by BLASTn. Sequences with ≤2 mismatches per primer (*E. amsterdamensis*, *E. enhydrae*) are expected to be amplified by the assay, so that a positive ER1F/ER1R PCR alone does not exclude these species; the amplicon sequence, however, separates them from *E. rhusiopathiae* (96.1–96.3% identity).

Accession	Organism	Strain	Host / source	Country	Year	ER1F mismatches	ER1R mismatches
AP012027.1	*Erysipelothrix rhusiopathiae*	Fujisawa	pig (serovar 1a reference)	Japan		0	0
AB019247.1	*Erysipelothrix rhusiopathiae*	ATCC 19414 (type strain)	pig			0	0
LR134439.1	*Erysipelothrix rhusiopathiae*	NCTC 8163	spleen			0	0
CP005079.1	*Erysipelothrix rhusiopathiae*	SY1027	pig	China		0	0
CP014861.1	*Erysipelothrix rhusiopathiae*	GXBY-1	swine	China: Guangxi	2012	0	0
CP017116.1	*Erysipelothrix rhusiopathiae*	WH13013	pig spleen	China: Wuhan	2013	0	0
CP029804.1	*Erysipelothrix rhusiopathiae*	ML101	pig	China: Miluo	2010	0	0
CP011860.1	*Erysipelothrix rhusiopathiae*	G4T10	swine	China	2013	0	0
CP011861.1	*Erysipelothrix rhusiopathiae*	SE38	swine	China	2013	0	0
CP080398.1	*Erysipelothrix rhusiopathiae*	B18		China: Hubei	2018	0	0
CP098031.1	*Erysipelothrix rhusiopathiae*	319078	human wound swab	Germany	2020	0	0
CP176524.1	*Erysipelothrix rhusiopathiae*	NL-11		South Korea		0	0
OZ373893.1	*Erysipelothrix rhusiopathiae*	AMX7	muskox carcass	Canada	2013	0	0
CP192437.1	*Erysipelothrix rhusiopathiae*	584	mallard duck	Poland	2021	0	0
CP184721.1	*Erysipelothrix rhusiopathiae*	147	domestic goose	Poland	2020	0	0
CP183044.1	*Erysipelothrix rhusiopathiae*	670	domestic goose	Poland	2020	0	0
CP180440.1	*Erysipelothrix rhusiopathiae*	1023	domestic goose	Poland	2021	0	0
CP180318.1	*Erysipelothrix rhusiopathiae*	1012	domestic goose	Poland	2020	0	0
CP033601.1	*Erysipelothrix rhusiopathiae*	KC-Sb-R1	rough-toothed dolphin (*Steno bredanensis*)	South Korea: South Sea	2018	1	1
CP125808.1	*Erysipelothrix rhusiopathiae* subsp. ohloneorum	UCD-4724-06	southern sea otter	USA: California	2006	0	0
CP096542.1	*Erysipelothrix sp.* Poltava	Poltava	backyard farm	Ukraine	2006	0	0
CP041995.1	*Erysipelothrix rhusiopathiae*	ZJ	pig	China: Sichuan	2016	0	0

**Accession**	**Organism**	**Strain**	**Host / source**	**Country**	**Year**	**ER1F mismatches**	**ER1R mismatches**
OW659496.1	*Erysipelothrix amsterdamensis*	A18Y020d	albatross chick carcass	Amsterdam Island		1	1
CP125807.1	*Erysipelothrix enhydrae*	UCD-4322-04	southern sea otter	USA: California	2004	1	2
CP152290.1	*Erysipelothrix sp.* P66	P66	Sunda pangolin (*Manis javanica*)	China	2019	2	4
CP144372.1	*Erysipelothrix piscisicarius*	THMN	human aortic valve	Thailand	2023	2	4
CP034234.1	*Erysipelothrix piscisicarius*	15TAL0474 (type strain)	tiger barb (*Puntigrus tetrazona*)	USA: Florida	2015	2	4
AB019250.1	*Erysipelothrix rhusiopathiae* (as deposited; *piscisicarius*-like)	715		Japan		3	4
AB019248.1	*Erysipelothrix tonsillarum*	ATCC 43339 (type strain)	pig tonsil	Japan		3	10
AB019249.1	*Erysipelothrix rhusiopathiae* (serovar 7; sequence identical to *E. tonsillarum*)	Pecs 56		Hungary		9	7
CP049859.1	*Erysipelothrix sp.* HDW6A	HDW6A	diving beetle (*Cybister brevis*)	South Korea	2019	13	5
CP092171.1	*Erysipelothrix sp.* D19-032	D19-032	smallmouth bass	USA	2019	12	10
CP013213.1	*Erysipelothrix larvae* (outgroup)	LV19	rhinoceros beetle larva	South Korea	2014	12	7

Table S2. Top BLASTn hits (NCBI core nucleotide collection, megablast) for the mitochondrial cytochrome b (1,143 bp) and control-region (480 bp) contigs obtained from the stranded dolphin (SL26-0209A).

Marker	Rank	Accession	Description	Query cover (%)	Identity (%)
cytochrome b	1	MH000365.1	*Delphinus delphis* voucher CRI007520, complete mitogenome (Korean waters)	100	100.00 (1,143/1,143)
cytochrome b	2–4	PP375177.1, PP375173.1, PP375171.1	*Delphinus delphis*, complete mitogenomes (USNM vouchers)	100	99.65
cytochrome b	59	PP761289.1	*Delphinus bairdii* voucher SBMNH2008-04, complete mitogenome (best *D. bairdii* hit)	100	98.95
cytochrome b	—	NC_060612.1; NC_012053.1	*Stenella frontalis* and *S. coeruleoalba*, complete mitogenomes (best *Stenella* hits at full query coverage)	100	97.90; 97.64
control region	1	MH000365.1	*Delphinus delphis* voucher CRI007520, complete mitogenome (Korean waters)	100	99.79 (479/480)
control region	2	KC295624.1	*Delphinus* sp. 1 AN-2013 haplotype KS0530, D-loop	100	99.38 (477/480)
control region	3	PP375146.1	*Delphinus delphis* voucher USNM:MAMM:571489, complete mitogenome	100	99.17 (476/480)
control region	9	KY212898.1	*Delphinus delphis* haplotype KH14, control region (East Sea, Korea; Cetacean Research Institute)	97	100.00 (465/465)
control region	11–12	AY046548.1, AY046546.1	*Stenella coeruleoalba* isolates NJNU0432 and NJNU0430, control region (unpublished direct submission, 2001)	97	100.00 (465/465)
control region	86	AY168600.1	*Stenella coeruleoalba*, D-loop (best *Stenella* hit at full query coverage)	100	98.75 (474/480)

Ranks refer to the position in the megablast hit list sorted by maximum score (core nucleotide collection, searched September 2026). The D-loop query is the 480-bp contig after removal of primer sequences and a low-quality 3′ terminal segment. Of the 100 best control-region hits, 96 were *Delphinus* records and four were records deposited as *Stenella coeruleoalba*; the two *S. coeruleoalba* records AY046546.1 and AY046548.1 (direct submissions from 2001) are identical to the query and to the East Sea *Delphinus delphis* haplotype KH14 over the 465 bp they share, whereas the *S. coeruleoalba* reference mitogenome (NC_012053.1) is 97.3% identical to the query. The control region alone therefore would not have excluded *S. coeruleoalba*, and the species assignment rests on cytochrome b, for which all 100 best hits were *Delphinus* and the best *Stenella* mitogenomes were ≤97.9% identical.

Table S3. Short-beaked common dolphin (*Delphinus delphis*) carcasses recorded by the Jeju stranding network between January 2024 and July 2026 (Jeju stranding records database, Whale–Marine Life Conservation Research Center, Gidang Institute of Marine Science, Jeju National University; unpublished data). During the same period 102 cetacean carcasses were recorded in total (2024, 35; 2025, 37; January–July 2026, 30), of which narrow-ridged finless porpoises accounted for 51. Live sightings, releases from set nets and entangled free-swimming animals are excluded. Pods of 50–100 common dolphins were sighted off Beomseom (Seogwipo) on 30 November 2024 and north-east of Biyangdo on 18 January 2025. Body lengths are the provisional field measurements recorded in the database at recovery (taken along the body contour); for the present case (SL26-0209A, 220 cm in the database) the straight-line length measured after thawing at necropsy was 214.3 cm (Section 2.2).

**Date**	**Location**	**Circumstance**	**Sex**	**Body length (cm)**
17 Dec 2024	Sinchang, Hangyeong-myeon (west coast)	Stranded	M	225
25 Dec 2024	Yongsu-ri, Hangyeong-myeon (west coast)	Stranded	–	–
26 Dec 2024	Sinchon-ri, Jocheon-eup (north coast)	Stranded	–	–
8 Oct 2025	Sinchang-ri, Hangyeong-myeon (west coast)	Stranded (species identification provisional)	M	200
11 Nov 2025	West of Chagwido (offshore)	Bycaught, longline fishery	M	150
5 Dec 2025	Suwon-ri, Hallim-eup (north-west coast)	Stranded	M	240
30 Dec 2025	Iho, Jeju-si (north coast)	Stranded	F	210
11 Jan 2026	Changcheon-ri, Andeok-myeon (south-west coast)	Stranded (present case, SL26-0209A)	M	220 (214 at necropsy)
14 Jan 2026	Hallim, Hallim-eup (north-west coast)	Stranded; suspected drift-net bycatch	F	–
18 Feb 2026	Sarabong, Jeju-si (north coast)	Stranded	F	220
28 Feb 2026	Bukchon-ri, Jocheon-eup (north coast)	Bycaught (dead)	M	235

Table S4. Alleles at the three positions at which the ER1F/ER1R amplicon sequence of the common dolphin SL26-0209A (PZ983794) differs from the *E. rhusiopathiae* type strain, in all *E. rhusiopathiae* genome assemblies available in the NCBI Assembly database (taxon 1648; accessed September 2026). Of 237 unique assemblies, 230 covered the three positions (36 contained the complete 356-bp inner amplicon; in 194 short-read drafts the locus lies at a contig break downstream of the rRNA operon, but all three positions are downstream of the break and were resolved); seven assemblies did not cover the region. Positions are given in the coordinates of the 356-bp inner amplicon (positions 262, 287 and 358 of the 400-bp product). The complete list of assemblies with accession, strain, host, country, year and allele calls is provided in Table S4.xlsx.

**Allele pattern (positions 240 / 265 / 336)**	**Assemblies (n)**	**Representative members**
A / C / T (type-strain pattern)	224	ATCC 19414, Fujisawa, NCTC 8163; all 18 isolates from the 2021 Dutch harbour-porpoise mortality; two bottlenose-dolphin isolates (USA, Alabama); four Korean pig genomes (WO, GC, KO, NL-11); 152 Australian pig isolates (EMAI collection)
A / C / C	5	KC-Sb-R1 (rough-toothed dolphin, Korea, 2018); EMAI_121 (pig, Australia, 2017); EMAI_7 (pig, Argentina, historical); EMAI_6 (pig, USA, historical); ERS3774393-megahit_004 (metagenome-assembled genome, Democratic Republic of the Congo, 2021)
A / A / T	1	E. rhusiopathiae subsp. ohloneorum UCD-4724-06 (sea otter, USA)
G / A / C (SL26-0209A)	0	Present case only
